# Solvent‐Free Dry‐Process Enabling High‐Areal Loading Selenium‐Doped SPAN Cathodes Toward Practical Lithium–Sulfur Batteries

**DOI:** 10.1002/smll.202503037

**Published:** 2025-04-07

**Authors:** Dong Jun Kim, Tae Hwa Hong, Jung Seok Lee, Hyun Wook Jung, Yoon Hak Lee, Han Young Jung, Hyeonji Jang, Jung Tae Lee

**Affiliations:** ^1^ Department of Convergent Biotechnology and Adavanced Materials Science Kyung Hee University 1732, Deogyeong‐daero, Giheung‐gu Yongin‐si Gyeonggi‐do 17104 Republic of Korea

**Keywords:** dry‐process, high‐areal loading, lithium‐sulfur batteries, MWCNT, SPAN cathode

## Abstract

In this study, a selenium‐doped sulfurized polyacrylonitrile (Se‐SPAN) cathode fabricated by a dry process with multi‐walled carbon nanotubes (MWCNT) and a polytetrafluoroethylene (PTFE) binder is proposed to address issues in currently developed dry‐processed cathodes. The dry‐processed Se‐SPAN (D/Se‐SPAN) is characterized by a dense, robust, and uniform structure that successfully resists the internal stress evolution caused by significant volume variations of the Se‐SPAN under high‐loading conditions. Understanding these architectural advantages in D/Se‐SPAN, the unrivaled potential of D/Se‐SPAN compared with traditional slurry‐processed Se‐SPAN cathodes (S/Se‐SPAN) is established through a series of in‐depth electrochemical‐mechanical investigations. As a result, the D/Se‐SPAN recorded ≈31.8 mAh cm^−2^ of reversible areal capacities under ultra‐high‐loading conditions (64.2 mg_Se‐SPAN_ cm^−2^) and exhibited remarkable cycle stability. Based on this study, vital design guidelines are provided for developing high‐loading S‐based dry cathodes crucial for realizing cost‐effective and eco‐friendly battery production.

## Graphical abstract

This study explores the D/Se‐SPAN cathode using a solvent‐free process, enhancing the electrochemical performance and structural stability of lithium‐sulfur batteries. By incorporating MWCNT and PTFE, the D/Se‐SPAN exhibits superior conductivity, high mass loading capability, and excellent long‐term cycling behavior compared to S/Se‐SPAN. This work provides valuable insights into the mechanisms behind dry electrode and its advantages over traditional slurry‐based method.

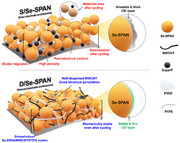

## 1 Introduction

Rechargeable batteries are ubiquitous and power a wide range of portable electronics, electric vehicles, and renewable energy systems. They are indispensable for a sustainable future, with research efforts focused on improving their energy density, cycle life, charging speed, and cost‐effectiveness.^[^ ^1^ ^]^ Recently, a dry process that eliminates the need for toxic organic solvents in battery electrode manufacturing has emerged as a promising approach, as it can simultaneously achieve these goals and provide additional benefits, such as reduced environmental impact, faster manufacturing, and improved scalability.^[^ ^2^ ^]^ The cost‐benefit primarily arises from the exclusion of the solvent evaporation and recovery stages, which naturally enhance environmental friendliness, manufacturing speed, and scalability. The enhanced energy density, fast charging capability, and cycle life are attributed to the facilitated electron transport via densified structures,^[^ ^3^ ^]^ introduction of 1D‐type conductive additives, and greater mechanical stability of the electrode, as opposed to those prepared via a slurry‐based process. The latter process induces “binder migration” and “1D carbon additive agglomeration” leading to the displacement of solid particles and the formation of cracks and voids.^[^ ^4^ ^]^

Nevertheless, challenges remain in the production of cathodes by the dry process, particularly regarding the components of the cathode materials. Traditional options such as polyanionic compounds (e.g., LiFePO_4_) or transition‐metal‐based layered oxide cathodes (e.g., LiCoO_2_, LiNi_x_Co_y_Al_1−x−y_O_2_, and LiNi_x_Co_y_Mn_1−x−y_O_2_), suffer from limited capacities, which restrict energy density. Moreover, the synthesis of these conventional cathode materials from lithium carbonate releases carbon dioxide, which contradicts the primary objective of reducing the environmental impact.^[^ ^5^ ^]^ As a result, further research is imperative to develop novel cathode materials that provide high capacity and have minimal environmental impact within the dry‐process framework. Therefore, sulfur (S)‐based cathodes with inherently high theoretical capacities present a highly promising solution to address the limitations of conventional materials. Additionally, the use of sulfur, a byproduct of petrochemical processes, aligns with environmental considerations and is cost‐effective. Acknowledging these advantages, several studies have explored the integration of S‐based cathodes with solvent‐free dry processes, aiming to develop a cathode with exceptional performance.^[^ ^6^ ^]^ However, S‐based cathodes produced through solvent‐free dry processes primarily encounter a critical limitation inherent to Li–S batteries: the dissolution and shuttle of lithium‐polysulfides (LiPSs) resulting in the loss of active material and a pronounced decline in capacity.^[^ ^7^ ^]^ Additionally, LiPSs react irreversibly with conventional carbonate‐based electrolytes, rendering them unusable and necessitating the adoption of more expensive and less safe ether‐based electrolytes.^[^ ^8^ ^]^ These issues present a major barrier to the practical implementation of dry‐processed S‐based cathodes, despite their considerable potential.

In addressing the key issues of current (S‐based) dry‐cathodes, in this study, we employed selenium‐doped sulfurized polyacrylonitrile (Se‐SPAN) as cathode materials to create high‐performance S‐based dry cathodes. SPAN‐based materials are recognized as highly promising materials for high‐loading S‐based cathodes‐crucial for the advancement of practical Li–S batteries owing to their distinctive electrochemical operation mechanisms, known as solid‐state conversion (SSC) mechanisms, different from those of conventional carbon/sulfur (C/S) composites.^[^ ^9^ ^]^ The SSC mechanism in SPAN‐based materials inherently prevents the formation of soluble LiPSs during the reaction, thus fundamentally overcoming the main challenges associated with high‐loading S‐based cathodes, as previously mentioned, and eventually enabling the use of conventional carbonate‐based electrolytes. In addition, the incorporation of a small amount of selenium into SPAN significantly enhanced its redox kinetics. Considering the remarkable potential of the Se‐SPAN cathode, we present a Se‐SPAN cathode fabricated by a dry process (D/Se‐SPAN) using multiwalled carbon nanotubes (MWCNT) and a polytetrafluoroethylene (PTFE) binder. In this configuration, we revealed that the utilization of a nonpolar MWCNT material facilitated the effective dispersion of relatively low‐polarity Se‐SPAN materials, resulting in the construction of a uniform, dense, and electrically well‐connected electrode architecture. The superior mechanical and electrical properties of the D/Se‐SPAN cathode led to superior electrochemical properties under high‐loading conditions, which were distinct from those of the Se‐SPAN cathode fabricated by a conventional slurry process (S/Se‐SPAN). By employing morphological analysis and tracking the variation in electrochemical impedance during constant cycling, we revealed that the loss of electrical percolation in the S/Se‐SPAN cathode was the main degradation factor in the formation protocol. In contrast, the high aspect ratio of the MWCNT enabled a robust electrode architecture in D/Se‐SPAN to maintain electrical percolating networks even when the active material content was increased to 95%. The structural and electrical integrity of D/Se‐SPAN enabled outstanding electrochemical performance in terms of rate capability, long‐term cyclability, and reversibility under high loading conditions compared to the reference S/Se‐SPAN cathode. Consequently, the synergy between Se‐SPAN and the dry‐process enabled D/Se‐SPAN to achieve remarkable cyclabilities over 300 cycles, maintaining ≈4.24 mAh cm^−2^ (0.38% decay per cycle) and reaching up to ≈31.8 mAh cm^−2^ of areal capacities under ultra‐high‐loading conditions (64.2 mg cm^−2^ of Se‐SPAN). This performance level is unprecedented for dry‐process‐based cathodes. Finally, the pouch‐type cell configuration with a reaching to practical conditions, such as high‐areal loading (12 mg_Se‐SPAN_ cm^−2^) and lean‐electrolyte conditions, exhibited 6.6 mAh cm^−2^ of areal capacities and maintained 95.6% of its capacity for 20 cycles. This study established the superior synergy between Se‐SPAN cathode materials and the solvent‐free dry process, providing a comprehensive design guideline for developing high‐loading S‐based cathodes crucial for realizing practical Li–S batteries.

## 2 Results and Discussion

### 2.1 Suitability of Se‐SPAN for Fabricating Dry‐Cathode

To develop SPAN‐based dry cathodes for practical applications, we focused on engineering active materials composed of polyacrylonitrile, sulfur, and an infinitesimal dose of Se as a dopant. The synthesis process and effect of Se doping were validated through a series of comprehensive analyses, including X‐ray photoelectron spectroscopy (XPS), thermogravimetric analysis (TGA), and electrochemical tests, as illustrated in Figure S1–S3 (Supporting Information). Incorporating selenium into SPAN is particularly important because it enhances the rate capability and accommodates high sulfur loading by accelerating the reaction kinetics and offering additional binding sites for sulfur within the pyrolyzed polyacrylonitrile matrix.^[^ ^10^ ^]^

**Figure 1:**
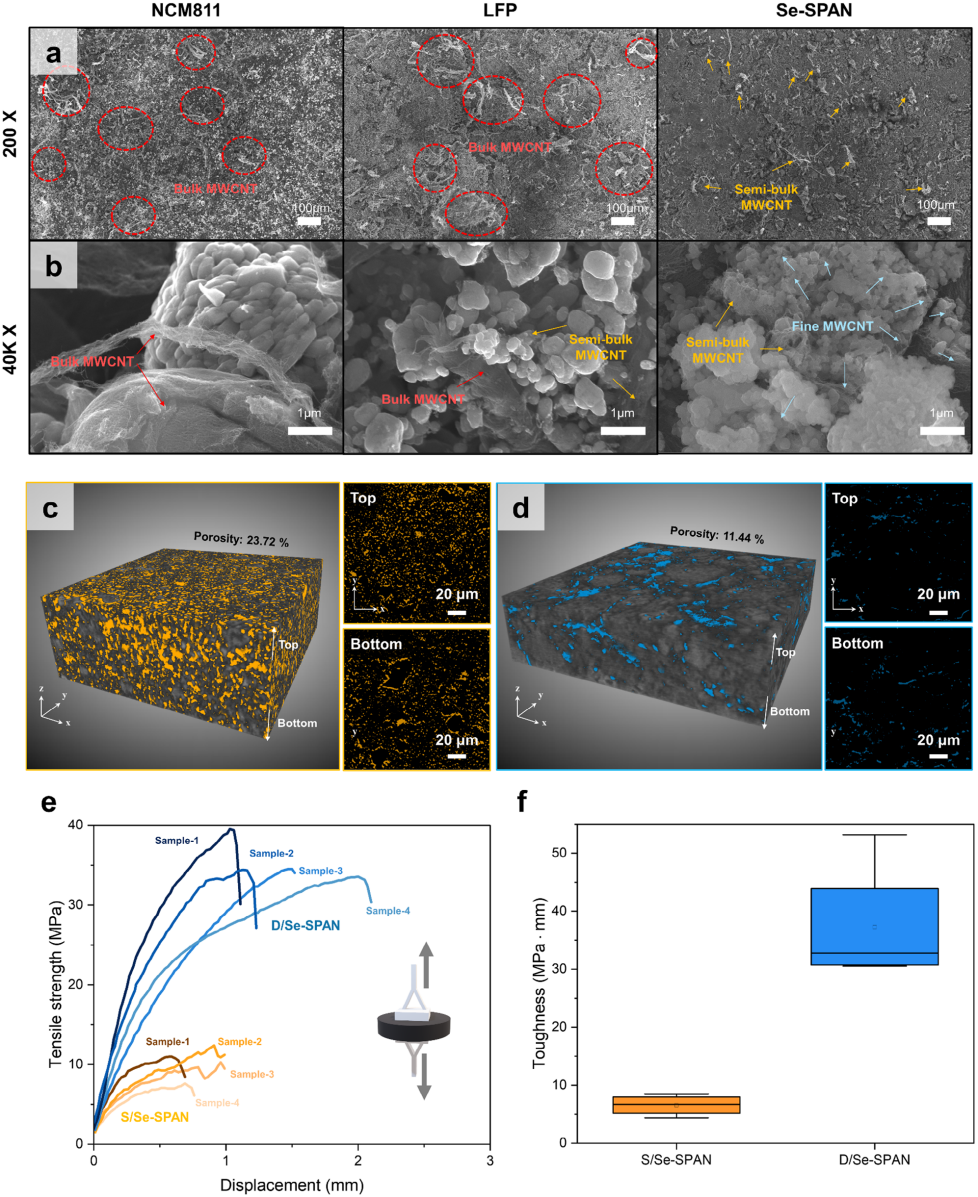
Structure and mechanical properties characterization for both S/Se‐SPAN and D/Se‐SPAN cathodes. SEM images depicting dispersion of MWCNT with different cathode materials for (a) 200 × magnification and (b) 40 K× magnification. 3D XRM images for (c) S/Se‐SPAN and (d) D/Se‐SPAN. Tensile strength test results for both cathodes, (e) stress‐strain behavior, and (f) toughness obtained by integration of stress curve.

**Figure 2:**
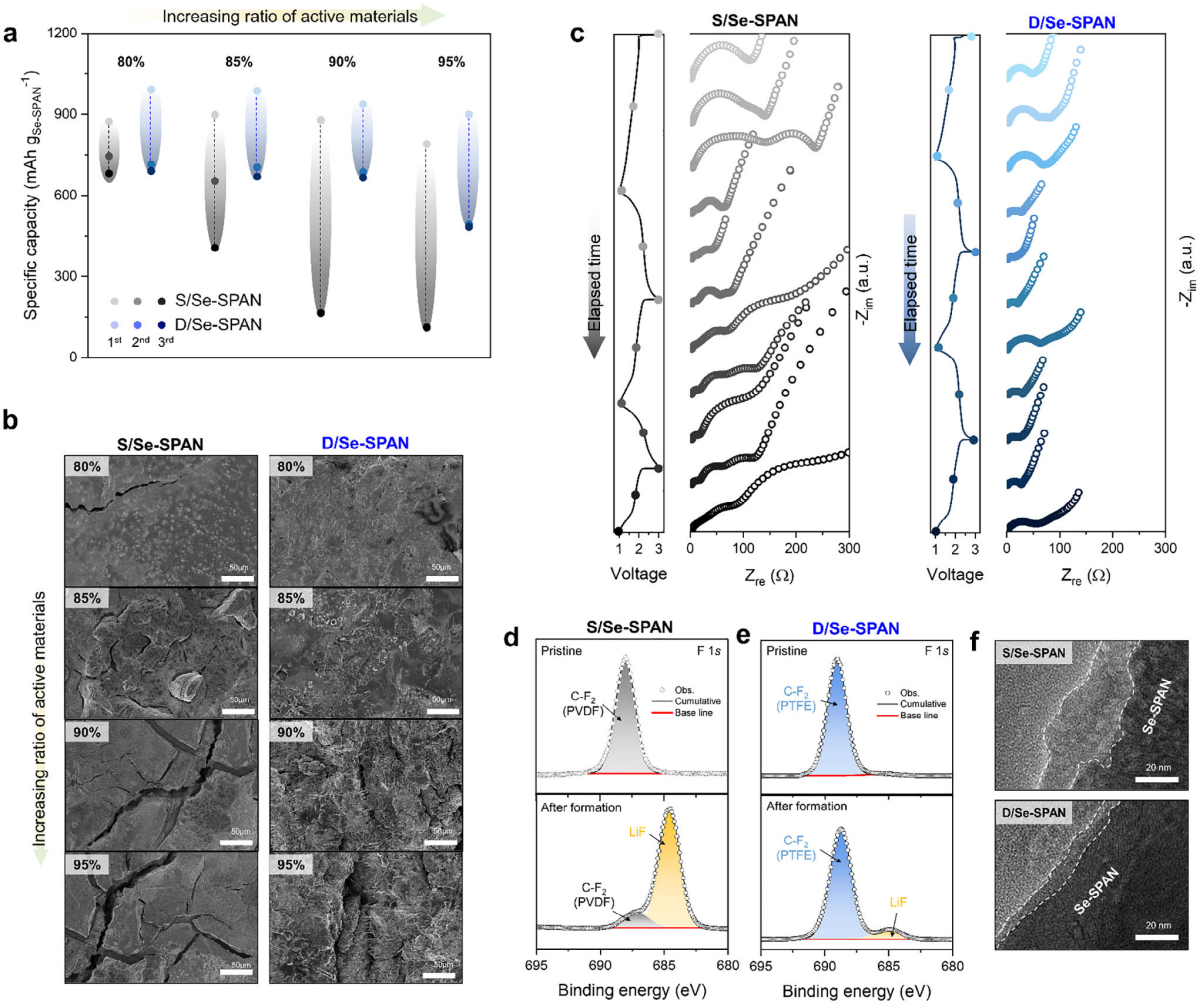
Electro‐chemo‐mechanical investigation for both cathodes during the formation protocol (initial three cycles). a) Obtained reversible capacity profiles during formation protocol. b) SEM images of the electrode surface for S/Se‐SPAN and D/Se‐SPAN after the formation protocol, categorized by different active material contents. c) EIS profiles tracking the formation protocol for both cathodes. F 1*s* XPS spectra obtained at the pristine state (upper panel) and after formation state (lower panel) for (d) S/Se‐SPAN and (e) D/Se‐SPAN cathodes. f) TEM images of S/Se‐SPAN and D/Se‐SPAN cathodes after formation protocol.

Using Se‐doped SPAN (Se‐SPAN) as the active material, we successfully developed robust high‐loading dry Se‐SPAN electrodes (denoted as D/Se‐SPAN), incorporating PTFE as the fibrous binder and MWCNT as the conductive agent (Figure S4, Supporting Information). The dry process facilitates the easy incorporation of high‐performance 1D‐type carbon additives such as CNT or VGCF, resulting in enhanced electrode performance.^[^ ^4e–g^ ^]^ This is attributed to–fact that in traditional slurry processes, 1D‐type carbon additives tend to aggregate in the solvent, whereas in the dry process, their agglomeration can be easily avoided because of the absence of a solvent. However, concerns persist regarding the uniform distribution of 1D‐type carbon additives in other electrode materials under solvent‐free conditions.^[^ ^11^ ^]^ In this regard, during the preparation of the dry electrode, we observed a remarkable mixability between the MWCNT and Se‐SPAN. This resulted in rapid dispersion of the MWCNT within the Se‐SPAN matrix. To delve deeper into this mixing compatibility under dry conditions, the mixing experiments were conducted with the high‐mass ratio of MWCNT (20 wt.%) and active materials (80 wt.%), including Se‐SPAN and conventional cathode materials such as Li(Ni_0.8_Co_0.1_Mn_0.1_)O_2_ (NCM811) and LiFePO_4_ (LFP). After a brief 2‐min mixing of the three cathode materials with MWCNT, we utilized high‐resolution field‐emission scanning electron microscopy (HR‐FE‐SEM) to examine the dispersity of the MWCNT within each cathode material (**Figure** **1**a). For NCM811 and LFP, agglomerated MWCNT sites are observed throughout the matrices. Conversely, the Se‐SPAN/MWCNT mixture showed significantly fewer agglomerated MWCNT sites, suggesting that Se‐SPAN facilitates rapid and uniform dispersion of MWCNT compared to conventional cathode materials. The mixing compatibility between the MWCNT and the active materials was further confirmed by high‐magnification (40 K×) SEM images, as depicted in Figure 1b. For the NCM811/CNT mixtures, only bulk MWCNT agglomerates are present on the active material particles, with no finely dispersed MWCNTs, indicating deficient dispersion and inadequate electrical connections. Similarly, bulk and semi‐bulk MWCNT agglomerates appeared in the LFP powders. However, the high‐magnification images of the Se‐SPAN/MWCNT mixtures revealed semi‐bulk MWCNT agglomerates alongside numerous finely split fibrils (denoted as fine MWCNT) covering the Se‐SPAN particles. These findings demonstrate the high compatibility of the MWCNT with the Se‐SPAN particles, resulting in finely split morphologies and well‐established electrical percolating networks between the particles. The enhanced MWCNT‐mixing compatibility in Se‐SPAN can be attributed to its relatively lower surface energy compared to other conventional cathode materials, as evidenced by contact angle measurements (Figure S5, Supporting Information). Given that MWCNT are known for their low surface energy, synergy with the relatively low surface energy of Se‐SPAN leads to both MWCNT and active materials (Se‐SPAN) exhibiting high mixing‐compatibility.^[^ ^12^ ^]^ Furthermore, the surface energy coherence between MWCNT and Se‐SPAN enables well‐dispersion,^[^ ^13^ ^]^ while the π‐conjugated interactions within the MWCNT and PAN matrices further enhance the dispersibility of MWCNT,^[^ ^14^ ^]^ ultimately leading to a more uniform conductive network. The correlation between the contact angle measurements and the MWCNT dispersity, as observed in the high‐magnification SEM images for the three types of cathode materials, substantiates this claim. This result reveals the inherently superior compatibility of the Se‐SPAN cathode materials with MWCNT and addresses the challenges associated with dispersing 1D‐type carbon materials into the cathode structure under solvent‐free conditions. This compatibility facilitates the development of high‐performance D/Se‐SPAN electrodes characterized by dense, robust, and electrically well‐connected architectures. To highlight the advantages of D/Se‐SPAN with MWCNT and PTFE binders over traditional slurry‐processed Se‐SPAN cathodes (denoted as S/Se‐PAN) that incorporate carbon black (SuperP) and PVDF binders, S/Se‐SPAN samples were prepared with a high areal loading comparable to that of D/Se‐SPAN (Se‐SPAN loading: 7 mg cm^−2^, active materials/binder/carbon additive (A/B/C) ratio: 8/1/1). A detailed examination using a 3D X‐ray microscope (XRM) revealed significant differences in the architectural features of both cathodes, including variations in pore size, porosity, pore connectivity, and pore distribution. S/Se‐SPAN cathode displayed poor electrode architecture, including high porosity (23.72%), nonuniformity (across top and bottom), and multiple voids particularly evident under high‐loading conditions (Figure 1c). The high porosity caused by the numerous voids formed during the solvent extraction drying process remains incomplete even after the high‐pressure calendaring process. The presence of solvent in the cathode manufacturing process triggers a phenomenon of binder migration within cast slurries, eventually resulting in an uneven distribution of electrode components (see the side panel in **Figure** **2**c).^[^ ^15^ ^]^ In contrast, D/Se‐SPAN cathodes exhibit voidless electrode architecture (11.44% of porosity) when compared with S/Se‐SPAN (Figure 1d). The solvent‐free conditions in the dry process and the established uniform Se‐SPAN/MWCNT matrices led to the absence of binder migration, which supports the fabrication of dense and uniform (across the top and bottom) electrode architectures (see the side panel in Figure 2d). Our claim on void contents between S/Se‐SPAN and D/Se‐SPAN is further verified with N_2_ adsorption/desorption analysis, as shown in Figure S6 (Supporting Information). This voidless architecture endows the D/Se‐SPAN with dense and robust mechanical properties, while also reducing electrolyte absorption for battery operation.^[^ ^9^, ^16^ ^]^ This results in a lower electrolyte‐to‐active materials (E/A) ratio, which is essential for the practical realization of Li–S batteries. The superior architectural features of the D/Se‐SPAN electrodes stemming from the uniform Se‐SPAN/MWCNT matrix coupled with the fibrillated PTFE binder were further confirmed through a series of mechanical tests. To evaluate the intrinsic adhesion strength of the electrode materials, we conducted pull‐off tests on the free‐standing S/Se‐SPAN and D/Se‐SPAN samples. Remarkably, the tensile strength of D/Se‐SPAN was approximately three times greater than that of S/Se‐SPAN (Figure 1e). This indicates that the interfacial bonding in the electrode matrix of D/Se‐SPAN was significantly stronger than that of S/Se‐SPAN. The superior physical properties of D/Se‐SPAN were further corroborated by its higher toughness, which was obtained by integrating the tensile tests (Figure 1f). The incorporation of PTFE and MWCNT in the D/Se‐SPAN enabled the electrode to absorb more energy before fracturing compared to the PVDF and SuperP in the S/Se‐SPAN. Nano‐indentation results (Figure S7a,b, Supporting Information) show that D/Se‐SPAN had a lower elastic indentation modulus (EIT), indicating higher flexibility and elasticity owing to the fibrous binder and conductive additive. The mechanical differences were further demonstrated through folding tests, which demonstrated the flexibility of each electrode (Figure S8a,b, Supporting Information). Consequently, D/Se‐SPAN demonstrated an enhanced ability to preserve the structural integrity and accommodate the volume changes of Se‐SPAN during charge/discharge cycles owing to its lower EIT and higher toughness. The exceptional mechanical properties of D/Se‐SPAN originating from its robust electrode architecture are expected to significantly enhance its electrochemical performance.

### 2.2 Origin of Capacity Reversibility in High‐Loading Se‐SPAN Cathodes

The dry process establishes a dense electron‐conductive network structure through 1D‐type carbon additives such as MWCNT and VGCF, significantly improving electrode performance with minimal dosage, thereby enabling high active material loading.^[^ ^17^ ^]^ To underscore the dry‐process, S/Se‐SPAN and D/Se‐SPAN were fabricated with active material ratios ranging from 80 to 95 wt.%. The ratio of carbon additive to binder is consistently maintained at 1:1, and the areal mass loading of Se‐SPAN is ≈7.0 mg_Se‐SPAN_ cm^−2^ for both cathodes. The S/Se‐SPAN containing 80 wt.% active materials exhibited an initial discharge capacity of 873.6 mAh g_Se‐SPAN_^−1^, retaining 681.7 mAh g_Se‐SPAN_^−1^ by the third cycle (Figure 2a). However, as the concentration of the active material increased, the reversible capacity rapidly decreased. Eventually, the S/Se‐SPAN cathodes containing 90 and 95 wt.% of the active materials showed almost no capacity maintenance. Research on SPAN‐based batteries, which utilize a solid‐state conversion mechanism, has revealed significant stress evolution during the charge/discharge process.^[^ ^18^ ^]^ This stress is further exacerbated by the defective electrode architecture of S/Se‐SPAN, which is characterized by numerous voids and an uneven material distribution. Such structural deficiencies render the electrodes highly susceptible to volume expansion during lithiation (discharging), which likely results in the loss of electrical percolation networks after several electrochemical reactions. Conversely, the D/Se‐SPAN electrodes demonstrate excellent reversible capacity maintenance over the initial three cycles, even when the active material ratio is increased to 90 wt.%. Specifically, the D/Se‐SPAN with 90 wt.% active material exhibits an initial capacity of 947.1 mAh g_Se‐SPAN_^−1^, and a reversible capacity of 675.0 mAh g_Se‐SPAN_^−1^ by the third cycle. Intriguingly, these values are comparable to those obtained with lower active material ratios, such as 80 wt.% (699.8 mAh g_Se‐SPAN_^−1^ in the third cycle) and 85 wt.% (678.7 mAh g_Se‐SPAN_^−1^ in the third cycle). These results suggest that the D/Se‐SPAN electrodes maintained consistent performance even with higher concentrations of the active material, showing minimal deviation in capacity compared to S/Se‐SPAN. The superior capacity reversibility of the D/Se‐SPAN, even with a low dose of conductive agents, can be attributed to the mechanical and electronic properties of the MWCNT and the robust electrode matrix reinforced by fibrillated PTFE. The robust electrode architecture of D/Se‐SPAN can resist stress during lithiation, with the 1D‐type MWCNT bridging the active materials even in the presence of cracks, thereby maintaining electrical percolation networks during electrochemical reactions.

SEM analysis of both electrodes after the third discharge revealed differences in the mechanical features of the cathodes, supporting our hypothesis. As shown in Figure 2b, cracks were observed in S/Se‐SPAN, indicating its inability to withstand the stress generated during the electrochemical reactions. The use of a spherical conductive additive with a low aspect ratio,^[^ ^17^, ^19^ ^]^ exacerbates this issue by hindering long‐range contact within multiple voids and cracks. This phenomenon rendered the S/Se‐SPAN electrodes susceptible to the loss of electrically percolating networks, leading to a decrease in capacity reversibility. Furthermore, as the proportion of active materials increased, the cracks became more severe, thereby worsening the adverse effects of S/Se‐SPAN. Consequently, the reversible capacity rapidly decreased with an increase in the number of active materials (reduced carbon additives), as shown in Figure 2a. On the other hand, almost all the D/Se‐SPAN electrodes exhibited crack‐free morphologies, benefiting from the robust and uniformly stable electrode matrix enabled by the Se‐SPAN/MWCNT network and fibrillated PTFE binder. This is clearly observed in cathodes consisting of 90–95 wt.% active materials, where the structure resists cracking due to significant internal stress and is supported by fibrous components, including MWCNT and fibrillated PTFE.

The evolution and variation in the structural/electrical properties of the electrodes during the initial cycling were thoroughly monitored via electrochemical impedance spectroscopy (EIS), providing in‐depth insights into their electrochemical behavior (Figure 2c). First, in the fresh state, D/Se‐SPAN exhibited a slightly lower charge transfer resistance (R_ct_) than S/Se‐SPAN, owing to the higher electronic conductivity of the MWCNT network compared to that of carbon black. During the 1^st^ discharge, both cathodes underwent an electrochemical lithiation process and the initiation of cathode‐electrolyte‐interphase (CEI) formation,^[^ ^20^ ^]^ as evidenced by the evolution of one distinct semicircle in the high‐frequency region (denoted as R_CEI_). Additionally, the prominent differences between S/Se‐SPAN and D/Se‐SPAN became more evident in subsequent cycles. For S/Se‐SPAN, three distinct semicircles appear during the second discharge process. The appearance of the last semicircle (denoted as R_int_) in the low‐frequency region signifies the detachment of the interfacial electrical contact^[^ ^21^ ^]^ between the conductive carbon agents and the Se‐SPAN particles. This R_int_ value continued to increase significantly with cycling, indicating the progressive formation of multiple cracks in S/Se‐SPAN, as previously revealed by SEM analysis (Figure 2b). In contrast, the D/Se‐SPAN, with its exceptional mechanical properties, withstood the volume variation of the Se‐SPAN particles without developing cracks. Additionally, the presence of 1D‐type MWCNT ensures excellent electrical percolation networks, thereby preventing the growth of R_int_. As a result, R_CEI_, R_ct_, and R_int_ values for S/Se‐SPAN after the third discharge are 21.7, 49.2, and 250.9 Ω respectively, while those for D/Se‐SPAN are 8.3, 30.0, and 55.5 Ω (Figure S9, Supporting Information). The lower R_ct_ and R_int_ values observed for D/Se‐SPAN suggest that its superior mechanical properties enable the preservation of electrode integrity and electrical connections, which S/Se‐SPAN failed to achieve. This is further demonstrated in Figure S10 (Supporting Information), where S/Se‐SPAN showed immediate percolation loss and delamination from the current collector after ultrasonic treatment following the third cycle. In contrast, D/Se‐SPAN maintained its structure without deformation owing to the mechanical strength provided by the MWCNT and PTFE, demonstrating excellent stress resistance.

The lower R_CEI_ value of the D/Se‐SPAN compared with that of the S/Se‐SPAN is also believed to stem from the distinct architectural and mechanical characteristics of the D/Se‐SPAN. To delve deeper into the characteristics of the CEI layer, we employed X‐ray photoelectron spectroscopy (XPS) and transmission electron microscopy (TEM) on both cathodes subjected to the formation protocol (three cycles under 0.05A g_Se‐SPAN_^−1^). Figure 2d shows the F 1*s* spectra of the pristine (upper panel) and pre‐cycled (lower panel) S/Se‐SPAN cathodes. Notably, for the precycled S/Se‐SPAN cathodes, we observed a distinct peak corresponding to LiF, which is the primary component of the CEI layer. Moreover, the peak attributed to C–F_2_ originating from the PVDF binder was significantly diminished. This reduction in the C–F_2_ peak in the precycled S/Se‐SPAN cathodes suggests encapsulation of the PVDF binder within S/Se‐SPAN by the thick CEI layer formed on the S/Se‐SPAN. Conversely, in the case of D/Se‐SPAN, our findings indicate a relatively weak LiF peak in the precycled D/Se‐SPAN cathodes compared with the pronounced C–F_2_ peak derived from the PTFE binder (Figure 2e). These disparities suggest that a thick CEI layer was formed on the S/Se‐SPAN cathodes, while a thinner CEI layer was formed on the D/Se‐SPAN cathodes during the formation protocol. The thicknesses of the CEI layers on both the cathodes were directly validated using TEM analysis, as shown in Figure 2f. A comparison of the observed CEI layers on both cathodes revealed that a thick and uneven CEI layer was formed on the surface of S/Se‐SPAN, whereas a thin and uniform CEI layer was formed on the surface of D/Se‐SPAN. These findings, supported by XPS and TEM analyses, strongly suggest that electrolyte decomposition and side reactions occurred at different levels in S/Se‐SPAN and D/Se‐SPAN during the formation. Based on our previous SEM and EIS studies, S/Se‐SPAN underwent significant fracturing of the electrode structure during the formation protocol. This fracturing leads to increased electrolyte decomposition and side reactions at the newly formed electrode‐electrolyte interfaces.^[^ ^22^ ^]^ This results in a thick and uneven CEI layer on S/Se‐SPAN. In contrast, D/Se‐SPAN, which withstood substantial stress during electrochemical reactions and maintained its original structure, experienced less electrolyte decomposition and fewer side reactions, thus forming a thin and uniform CEI layer. These disparities not only contribute to capacity degradation during the initial cycling, but also impact the overall performance of the cell owing to differences in the constructed CEI layer. The discovery of high‐capacity reversibility in high‐loading D/Se‐SPAN cathodes during the initial formation protocol, which was studied through a series of comprehensive electrochemomechanical analyses of both cathodes, indicates that D/Se‐SPAN exhibits significantly superior electrochemical performance compared to conventional slurry‐processed Se‐SPAN cathodes.

### 2.3 Superior Electrochemical Performance of D/Se‐SPAN over S/Se‐SPAN

**Figure 3:**
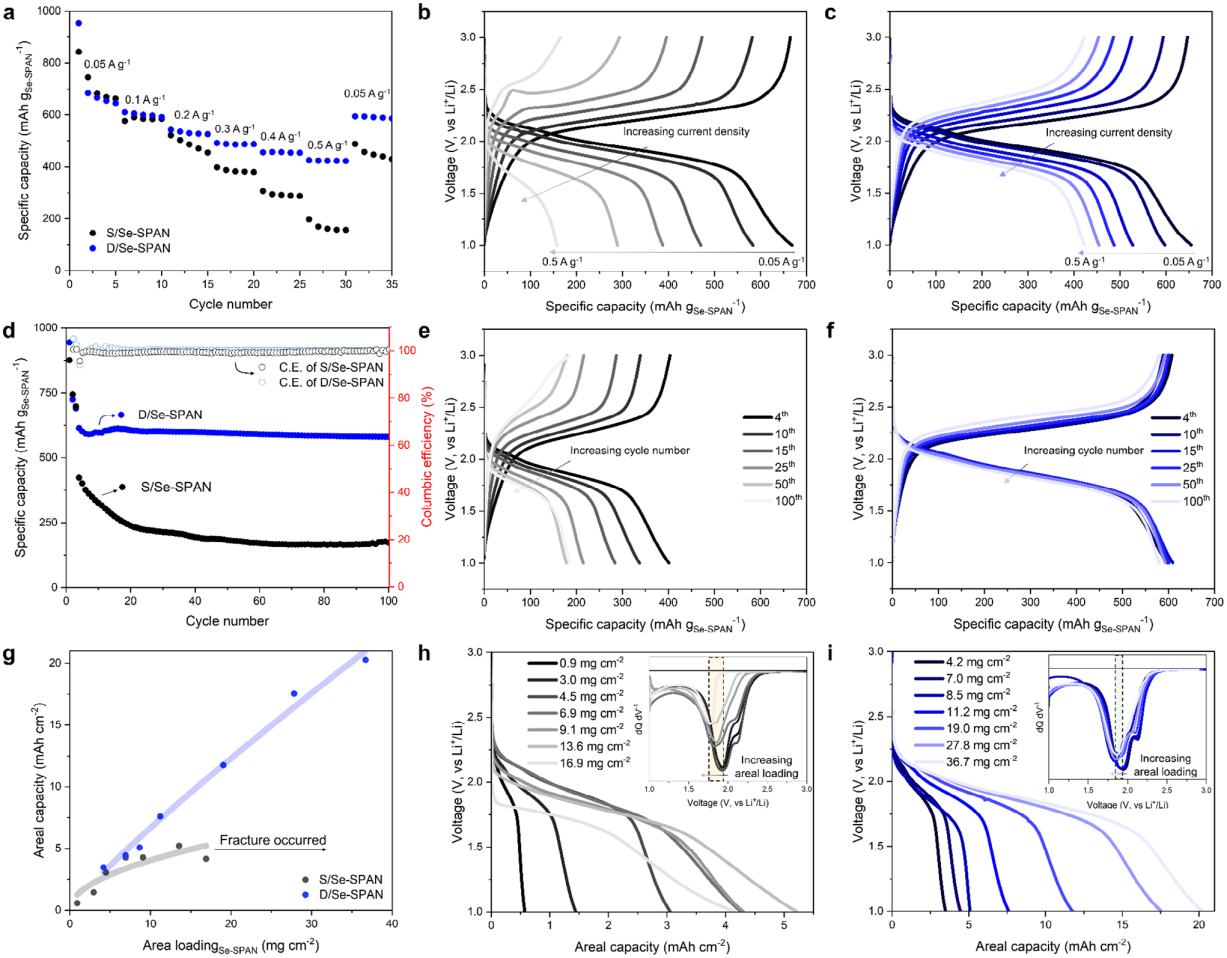
Electrochemical performance comparison between S/Se‐SPAN and D/Se‐SPAN cathodes. a) Specific capacity profiles for both cathodes under increasing current densities. Voltage profiles at various current densities for (b) S/Se‐SPAN and (c) D/Se‐SPAN. d) long‐term cycle behavior for both cathodes. Voltage profiles with increasing cycle number for (e) S/Se‐SPAN and (f) D/Se‐SPAN. g) Areal capacity profiles with increasing Se‐SPAN loading for both cathodes. Discharge voltage profiles with increasing Se‐SPAN loadings for (h) S/Se‐SPAN and (i) D/Se‐SPAN. The dQ dV^−1^ profile corresponding to discharge curves were presented in the inset.

The outstanding structural, mechanical, and electrical properties of D/Se‐SPAN enhanced the electrochemical performance of Li–S cells, particularly in terms of rate capability, long‐term cyclability, and reversibility under ultrahigh‐loading conditions. **Figure** **3**a illustrates the rate‐capability profiles of both electrodes with an areal loading of ≈5.0 mg_Se‐SPAN_ cm^−2^ at current densities ranging from 0.05 A g_Se‐SPAN_^−1^ to 0.5 A g_Se‐SPAN_^−1^. In a comprehensive overview, D/Se‐SPAN demonstrates higher capacities than S/Se‐SPAN across all current densities. Notably, when the current density is reduced back to 0.05 A g_Se‐SPAN_^−1^ from 0.5 A g_Se‐SPAN_^−1^, D/Se‐SPAN maintains superior electrochemical stability, exhibiting a specific capacity of 589.5 mAh g_Se‐SPAN_^−1^ compared to 440.0 mAh g_Se‐SPAN_^−1^ in S/Se‐SPAN. Delving deeper, S/Se‐SPAN exhibits specific capacities of 668.7, 584.0, 470.5, 381.24, 289.5, and 157.5 mAh g_Se‐SPAN_^−1^ under current densities ranging from 0.05 to 0.5 A g_Se‐SPAN_^−1^ respectively, with showing rapidly increasing voltage hysteresis dependent on current densities, as illustrated in Figure 3b. D/Se‐SPAN, while initially displaying similar performance to S/Se‐SPAN at low current densities (0.1 A g_Se‐SPAN_^−1^), distinguishes itself by maintaining excellent discharge capacities, eventually achieving a high reversible capacity of 422 mAh g_Se‐SPAN_^−1^ under harsh conditions of 0.5 A g_Se‐SPAN_^−1^. Furthermore, the rate‐dependent voltage hysteresis was significantly reduced compared with that of S/Se‐SPAN, as depicted in Figure 3c. The superior properties of D/Se‐SPAN, particularly under high loading conditions, contributed to its lower overall cell resistance and significantly improved rate‐dependent performance compared to S/Se‐SPAN, even when examined under identical areal loading conditions.

The exceptional characteristics of the D/Se‐SPAN cathodes also enhanced their long‐term cycling performance. As illustrated in Figure 3d, cycling tests conducted on both S/Se‐SPAN and D/Se‐SPAN cathodes under a current density of 0.2 A g_Se‐SPAN_^−1^ post‐formation cycle (preceded by 0.05A g_Se‐SPAN_^−1^ for three cycles) reveal noteworthy differences. D/Se‐SPAN exhibits high initial capacities, starting at 614.6 mAh g_Se‐SPAN_^−1^ and maintains a 580.1 mAh g_Se‐SPAN_^−1^ reversible capacity even after 100 cycles. In contrast, S/Se‐SPAN displayed poorer cycling properties, with the capacity declining from 423.3 mAh g_Se‐SPAN_^−1^ to 173.3 mAh g_Se‐SPAN_^−1^ after 100 cycles. Furthermore, D/Se‐SPAN demonstrated stable charge/discharge voltage profiles over 100 cycles, whereas S/Se‐SPAN exhibited instability in its voltage profiles by the 100th cycle, as depicted in Figure 3e,f. The D/Se‐SPAN cathodes maintained their original architecture during long‐term cycling because of their mechanical stability, whereas the instability of S/Se‐SPAN led to increased cracking during the cycling experiments (Figures S11 and S12, Supporting Information). Moreover, the increased surface area resulting from gradual electrode cracking in S/Se‐SPAN led to the breakdown of the existing CEI layer and the formation of a new CEI on the newly exposed surfaces. This process ultimately resulted in a thicker CEI, which significantly increased the resistance and accumulation of dead active materials, leading to a rapid decline in capacity during cycling tests. In contrast, the stable D/Se‐SPAN demonstrated notable capacity retention over 100 cycles (Figure S13, Supporting Information).

The synergistic combination of Se‐SPAN and the dry process enables high‐capacity reversibility even under high areal loading by mitigating the significant internal stresses induced by harsh conditions. Figure 3g shows the tracked reversible capacities of S/Se‐SPAN and D/Se‐SPAN under increasing areal loading conditions. For S/Se‐SPAN, a quasi‐linear correlation is observed between areal mass loading and areal capacities, spanning from low loading (0.9 mg_Se‐SPAN_ cm^−2^, yielding 0.57 mAh cm^−2^) to high loading (9.1 mg_Se‐SPAN_ cm^−2^, yielding 3.45 mAh cm^−2^). However, beyond the 9.1 mg_Se‐SPAN_ cm^−2^ area loading, the areal capacities and corresponding voltage profiles (see Figure 3h and dQ dV^−1^ profiles suggested in the inset) rapidly decayed, mainly because of the extreme exacerbation of its poor structural properties under harsh high‐areal loading conditions. In addition, the Se‐SPAN cathodes produced via the slurry process with much higher loadings displayed severe electrode cracking and delamination, rendering them unusable (Figure S14, Supporting Information). In contrast, D/Se‐SPAN maintains a linear relationship up to ultra‐high loadings of 36.7 mg_Se‐SPAN_ cm^−2^, yielding a high reversible areal capacity of 20.2 mAh cm^−2^. Furthermore, the voltage profiles of D/Se‐SPAN remained almost consistent with increasing areal loading, highlighting the highly stable electrochemical behavior of D/Se‐SPAN, even under much higher areal loading conditions (see Figure 3i and the dQ dV^−1^ profiles in the inset). These findings highlight the superior electrode properties of the D/Se‐SPAN, stemming from the synergistic combination of the Se‐SPAN/MWCNT matrix and fibrillated PTFE, which enables it to withstand extreme internal stresses induced by harsh loading conditions.

### 2.4 Mechanism for the Enhancement of High‐Areal Loading Se‐SPAN Cathode Fabricated by the Dry Process

**Figure 4:**
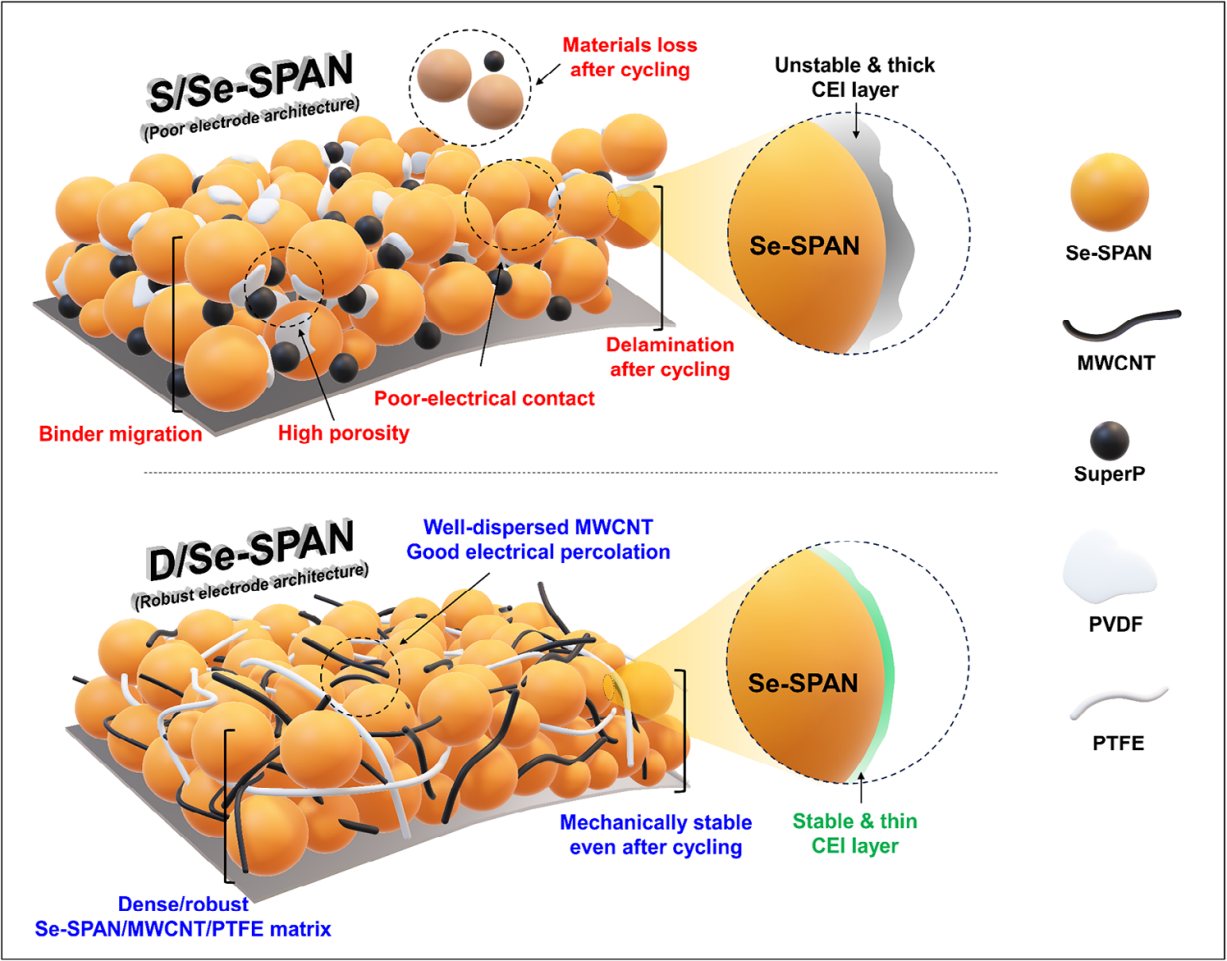
Schematic illustration of the enhancing mechanisms in high‐areal loading Se‐SPAN cathode fabricated by dry‐process with MWCNT and PTFE binder.

A series of experimental studies thoroughly elucidated the mechanism behind the superior structural and electrochemical performance of high‐areal‐loading Se‐SPAN cathodes fabricated by a dry process in comparison to those based on the conventional slurry process. In summary, during the solvent evaporation process for S/Se‐SPAN, binders that initially adhere to the electrode materials can migrate or be displaced, resulting in uneven distributions around the solid particles. This could lead to the formation of irregular pore structures and voids within the electrode, resulting in an unstable electrode architecture. In certain regions, the excessive accumulation of binders can obstruct the flow of electrical pathways, whereas in other areas, insufficient binders can cause structural instability. These structural features of S/Se‐SPAN have the potential to negatively impact the performance of batteries, as summarized in the upper panel of **Figure** **4**. Repeated electrochemical reactions of S/Se‐SPAN led to reduced electrical connectivity and mechanical stability, ultimately causing capacity degradation. In contrast, the D/Se‐SPAN utilizing a fibrillated PTFE binder and MWCNT possesses several advantages over the S/Se‐SPAN, as summarized in the lower panel of Figure 4. Specifically, D/Se‐SPAN involves directly combining electrode materials without the use of solvents, therefore this approach eliminating the issue of binder migration. In addition, Se‐SPAN underscores its high compatibility with dispersing MWCNT, which allows for the effective utilization of high‐performance MWCNT. In addition, the fibrous structure of PTFE in the D/Se‐SPAN differs from the way PVDF coats the surface of the active materials, forming bridges between particles.^[^ ^23^ ^]^ This configuration can lead to a decrease in the overall resistance within the electrode and enhance its performance. The synergistic combination of solvent‐free conditions, uniform Se‐SPAN/MWCNT matrices, and incorporation of a fibrillated PTFE binder resulted in D/Se‐SPAN exhibiting superior structural, mechanical, and electrical properties compared to S/Se‐SPAN. This configurational feature of D/Se‐SPAN allows to itself has many advantages for a highly stable electrochemical performance, as summarized in Figure 4.

### 2.5 Toward Practical High‐Performance Li–S Batteries with D/Se‐SPAN

**Figure 5:**
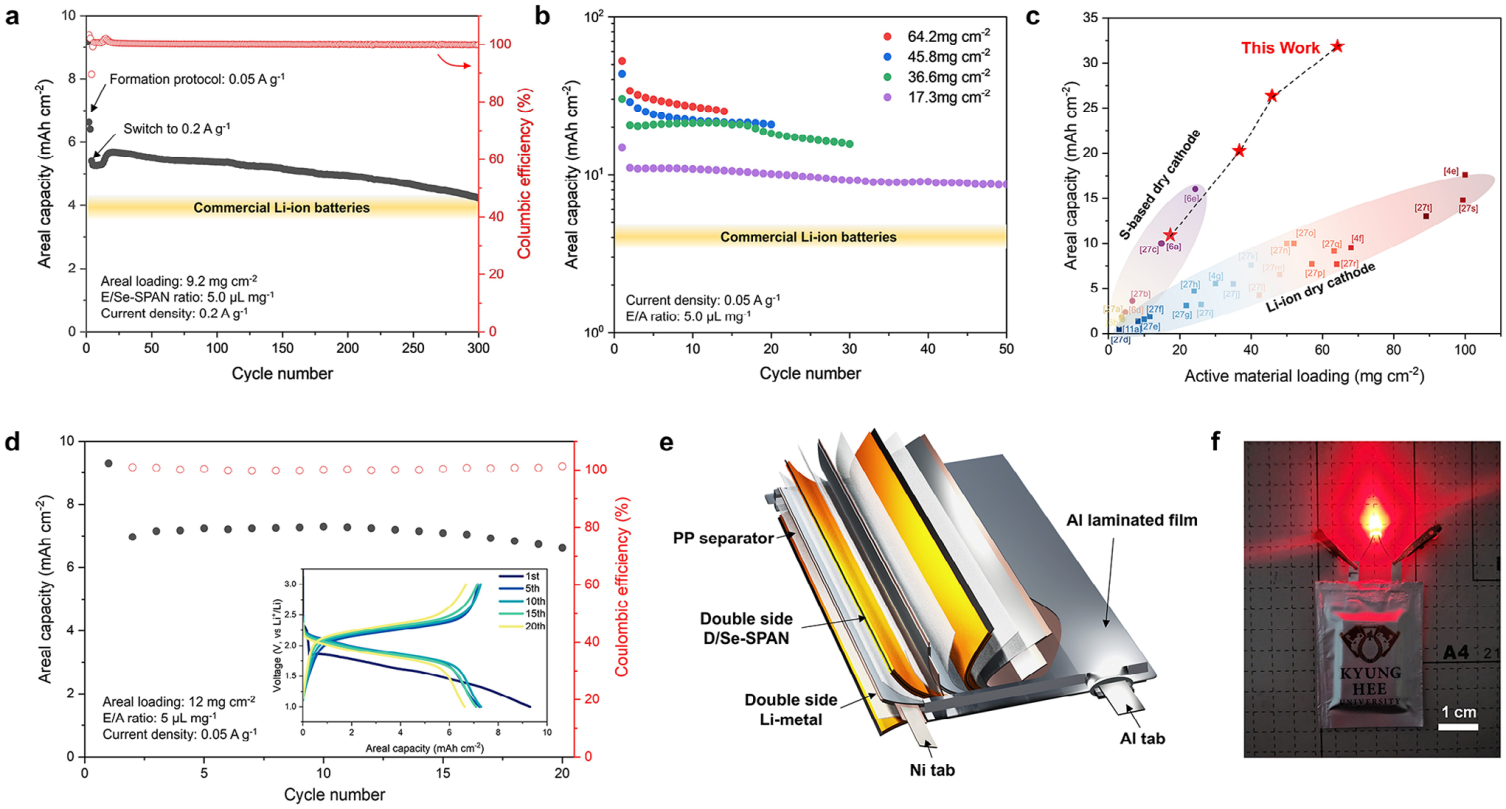
Electrochemical performances of ultra‐high areal loading D/Se‐SPAN cathode under practical conditions. a) Charge/discharge cycling test of D/Se‐SPAN with high areal loading (9.2 mg_Se‐SPAN_ cm^−2^) with the current density of 0.2 A g_Se‐SPAN_^−1^. b) Cycle behaviors for D/Se‐SPAN under ultra‐high areal loading conditions with the current density of 0.05 A g_Se‐SPAN_^−1^. c) Comparison of active materials loading versus areal capacity profiles between this work and reported cathode fabricated by dry‐based technology. d) Electrochemical charge/discharge behavior for single‐layered D/Se‐SPAN pouch cell under practical conditions. e) Schematic illustration for the five‐layer‐stacked pouch battery based on the double‐layer coated D/Se‐SPAN cathodes. f) demonstration for successful operation of 5‐stacked D/Se‐SPAN pouch battery.

To highlight the practical potential of D/Se‐SPAN, we evaluated the cycle life of batteries under high‐loading conditions in both coin and pouch cells under lean‐electrolyte conditions (E/A ratio: ≈5.0 mL g^−1^). First, D/Se‐SPAN with a high areal loading of 9.2 mg_Se‐SPAN_ cm^−2^ demonstrated a high initial areal capacity of 5.4 mAh cm^−2^ surpassing commercial areal capacities (≈4 mAh cm^−2^), as depicted in **Figure** **5**a, and also maintained 78.4% of its capacity over 300 cycles, with an average capacity decay of 0.38% per cycle. The primary degradation factor in the D/Se‐SPAN cell stems from the unstable Li deposition behavior and electrolyte consumption during formation of the SEI layer on the Li metal. This is demonstrated by the more pronounced degradation observed in the charging plateau than in the discharging plateau (see the voltage profiles depicted in Figure S15, Supporting Information). Consequently, enhancing the electrochemical stability of the Li‐metal anode through appropriate strategies, such as constructing a desirable SEI by electrolyte engineering,^[^ ^24^ ^]^ employing artificial coating agents on lithium metal surface,^[^ ^25^ ^]^ and micronizing Li deposition behaviors with pore regulation of the separator,^[^ ^26^ ^]^ could result in notable improvements in the overall cycling performance of the battery. The D/Se‐SPAN cells demonstrated superior cycling properties even under higher areal loading conditions. Remarkably, even at harsh areal loading levels up to 64.2 mg_Se‐SPAN_ cm^−2^, D/Se‐SPAN maintained high reversibility throughout cycle operations (Figure 5b). To the best of our knowledge, these results establish a world‐record for ultra‐high areal capacities in cathode materials, achieved through dry‐process techniques, including binder‐fibrillation, spray coating, and hot‐press protocols (Figure 5c).^[^ ^4^, ^6^, ^11^, ^27^ ^]^ Finally, we fabricated a homemade single‐layer pouch‐type cell utilizing 2.0 × 3.0 cm^2^ sized D/Se‐SPAN cathodes, with a high‐areal loading of 12 mg_Se‐SPAN_ cm^−2^, achieving high‐areal capacities of 7 mAh cm^−2^. Electrochemical measurements on the pouch cell at 0.05 A g_Se‐SPAN_^−1^ showed stable areal capacities of 6.6 mAh cm^−2^ over 20 cycles (Figure 5d). Notably, despite the high areal loading and wide reaction area, D/Se‐SPAN exhibited superior electrochemical stability with minimal voltage decay, as illustrated in the inset of Figure 5d. In addition, to demonstrate the feasibility of high‐loading Se‐SPAN cathodes using dry‐processing techniques, a double‐sided dry‐coating process^[^ ^28^ ^]^ was employed to fabricate multilayer pouch cells, as illustrated in Figure S16 (Supporting Information). Ultimately, the successful fabrication and operation of a five‐layer stacked pouch cell based on double‐side‐coated D/Se‐SPAN highlights its potential for advancing practical Li–S batteries (Figure 5e,f).

## 3 Conclusion

In this study, we developed high‐performance sulfur‐based cathodes (termed D/Se‐SPAN) using an environmentally friendly solvent‐free dry process with 1D‐type MWCNT as a conductive additive and fibrillated PTFE as a binder. We successfully established excellent mixing compatibility between the SPAN and MWCNT, highlighting Se‐SPAN as a promising candidate for high‐performance dry cathodes. The synergy between this compatibility and the solvent‐free conditions enables uniform, robust, and electrically well‐connected structures, surpassing those of the slurry‐processed Se‐SPAN cathode (S/Se‐SPAN) that uses the SuperP carbon additive and PVDF binder. The investigation of both cathodes during the initial formation protocols revealed that the superior mechanical properties of D/Se‐SPAN supported its high electrochemical reversibility against mechanical and electrical failures owing to electrode fatigue. Consequently, D/Se‐SPAN exhibited superior electrochemical performance in terms of rate capability, cyclability, and reversibility under high loading conditions compared to S/Se‐SPAN. Notably, we demonstrated its superior cycling performance of up to 300 cycles, ultra‐areal loading/capacity of up to 64.2 mg_Se‐SPAN_ cm^−2^, areal capacity of 31 mAh cm^−2^, and successful operation of pouch‐type batteries under lean‐electrolyte conditions. The potential of D/Se‐SPAN demonstrated in this study paves the way for high‐performance sulfur‐based cathodes, offering a promising avenue for cost‐effective and environmentally friendly energy storage solutions.

## Conflict of Interest

The authors declare no conflict of interest.

## Author Contributions

D.J.K. and T.H.H. contributed equally to this work.

## Supporting information



Supporting Information

## Data Availability

The data that support the findings of this study are available from the corresponding author upon reasonable request.
