# Supplementary material for: Solvent‐Free Dry‐Process Enabling High‐Areal Loading Selenium‐Doped SPAN Cathodes Toward Practical Lithium–Sulfur Batteries
Source: Small. 2025 Apr 7;21(22):2503037. doi: 10.1002/smll.202503037 (PMC12138843; doi:10.1002/smll.202503037)
Supplement: Supplementary file 1 — Supporting Information [file SMLL-21-2503037-s001.docx]

Supporting Information

**Solvent-free dry-process enabling high-areal loading selenium-doped SPAN cathodes toward practical lithium–sulfur batteries**

Dong Jun Kim,^‡^ Tae Hwa Hong,^‡^, Jung Seok Lee, Hyun Wook Jung, Yoon Hak Lee, Han Young Jung, Hyeonji Jang and Jung Tae Lee*

**Experimental Methods**

***Synthesis of SPAN and Se-SPAN***

Poly(acrylonitrile) (PAN weight-averaged molecular mass Mw = 150,000, Aldrich), elemental sulfur (99.8% metal trace), and selenium (99.9% metal trace, Sigma Aldrich) were homogeneously mixed in a mortar at a weight ratio of 1:3: 0.6 respectively. The mixed powder was placed in an alumina boat and heated in a tube furnace at 350 °C for 4 hours under N_2_ atmosphere. Subsequently, at 250 °C, the remaining sulfur on the surface was exposed to a flow of N_2_ for 3 h to remove it, resulting in the formation of the Se-SPAN composite material. When selenium was omitted, the process yielded a SPAN composite material.

***Electrode preparation***

The Se-SPAN dry cathode (D/Se-SPAN) was fabricated using a solvent-free process consisting of three steps. First, dry powders of Se-SPAN and multi-walled carbon nanotubes (MWCNT) were pre-mixed using a mortar in a weight ratio of Se-SPAN:MWCNT = 80:10 (or 85:7.5, 90:5, 95:2.5), followed by blending PTFE (Teflon PTFE 601X, Chemours Product) powder in the same weight ratio as MWCNT to serve as a binder. The mixed powders were homogenized using a high-speed mixer for uniform powder blending. Second, through PTFE kneading, the mixed powder was prefabricated into a free-standing electrode film. Finally, the free-standing electrode was calendared using a hot rolling machine at 80 ℃ and laminated onto a carbon-coated aluminum current collector. The D/Se-SPAN mass loading of the active material ranged from 5 mg_Se-SPAN_ cm^-2^ to 64.2 mg_Se-SPAN_ cm^-2^.

The S/Se-SPAN electrode was fabricated using a conventional wetting process. A slurry was prepared using a thin mixer to homogeneously disperse Se-SPAN, Super P, and PVDF in weight ratios of 80:10:10 (85:7.5:7.5, 90:5:5, and 95:2.5:2.5) in N-methyl-pyrrolidone (NMP). The Se-SPAN slurry was cast onto a carbon-coated aluminum current collector using a doctor blade. Subsequently, the slurry electrodes were vacuum-dried at 100 °C overnight. After drying, the electrodes were subjected to a roll-press calendar.

**Electrochemical characterization**

The D/Se-SPAN and S/Se-SPAN electrodes were cut into disks with diameters of 10 mm for coin-cell testing. The Se-SPAN electrode was assembled into a 2023-type coin cell using a Celgard 2400 separator, a Li metal counter electrode, and 1 M LiPF_6_ (EC/DEC/DMC=1:1:1, v/v/v) electrolyte with 10 wt% fluoroethylene carbonate (FEC) in an Ar-filled glove box (MOTEK, O_2_, H_2_O < 0.1ppm). For pouch cells, the D/Se-SPAN electrode was prepared in dimensions of 2 × 3 cm^2^ (areal loading = 12 mg_Se-SPAN_ cm^-2^), along with Celgard 2400 separator, Li metal counter electrode, and 1 M LiPF_6_ (EC/DEC/DMC=1:1:1, v/v/v) electrolyte with 10 wt% FEC. Coin cell electrochemical tests were performed at room temperature using a Neware Battery System (NMW-200-160CH). Before testing, the assembled coin cell was allowed to rest for 12 h, during which the cathode was soaked in the electrolyte. Galvanostatic charge–discharge tests were performed within the 1.0–3.0 V (vs. Li^+^/Li) voltage range. Rate capability tests were conducted at various current rates: 0.05, 0.1, 0.2, 0.3, 0.4 and 0.5A g_Se-SPAN_^-1^. Cyclability tests were performed at 0.2 A g_Se-SPAN_^-1^ after a formation cycle (at 0.05 A g_Se-SSPAN_^-1^ for 3 cycles). In addition, cyclability tests with the high-loading D/Se-SPAN electrodes were conducted at a current density of 0.05 A g_Se-SPAN_^-1^.

Electrochemical Impedance Spectroscopy (EIS) was performed using a CV ZIVE sp1 instrument (WonATech). EIS measurements—taken at the initial discharge to 1.7 V, charge to 2.2 V, and subsequent discharge to 1.9 V—were conducted during the formation of both S/Se-SPAN and D/Se-SPAN electrodes. Additionally, EIS measurements were performed for both cells at the formation stage and after 100 cycles to investigate the changes that occurred during the cycling process.

**Materials characterization**

TGA (Thermogravimetric Analysis) and X-ray photoelectron spectroscopy (XPS) were employed to confirm the Se doping of the SPAN. Thermogravimetric analysis (TGA) was performed using a TGA Q5000 IR instrument under a nitrogen atmosphere, where the temperature was increased to 1000 °C at a rate of 10 °C min^-1^. The XPS analysis was conducted using a K-alpha spectrometer (Thermo Scientific) with an Al K-alpha radiation source and a resolution of 0.1 eV. A high-resolution field-emission scanning electron microscope (HR FE-SEM; MERLIN, Carl Zeiss) was employed to examine the impact of mixing with MWCNTs on the dispersion of NMC811, LFP, and Se-SPAN, as they were mixed for 2 min, which is the same duration as that of MWCNTs. Additionally, the surfaces of S/Se-SPAN and D/Se-SPAN electrodes were observed after three charge–discharge cycles with active material compositions of 80%, 85%, 90%, and 95%. 3D X-ray microscopy (XRM) was used to investigate the morphological characteristics of the S/Se-SPAN and D/Se-SPAN, providing high-resolution three-dimensional imaging with a resolution of 0.3 µm. The nanoindentation tests were performed using a CPX-NHT2 to evaluate the mechanical properties of both cathodes. The experiments were conducted with a maximum load of 20 mN and a loading/unloading rate of 40 mN s^-1^. During testing, a continuous load was applied to the sample until it reached the maximum load, maintained for 5 s, followed by a gradual reduction at the same rate. The load and displacement were continuously monitored and recorded throughout the test to obtain the load-displacement data, which were subsequently used to determine the elastic modulus of the sample using the Oliver-Pharr method. In the pull-off test, both the S/Se-SPAN and D/Se-SPAN electrodes were fabricated into free-standing disks with a diameter of 1 cm using a Texture Analyzer (CT3 25 K, Brookfield). Tapes (0.5 × 0.5 cm^2^) were attached to both sides of each electrode, with the test conducted by applying a pull-off force to evaluate the internal adhesion properties of the electrodes. The differences between the CEI were characterized using field-emission transmission electron microscopy (FE-TEM; JEM-2100F, JEOL) and XPS. All post-cycling analyses were conducted using a vacuum chamber for transportation from the argon-filled glove box to the instrument chamber to minimize exposure to H_2_O and O_2_. This procedure ensured that the samples remained uncontaminated for subsequent analysis.


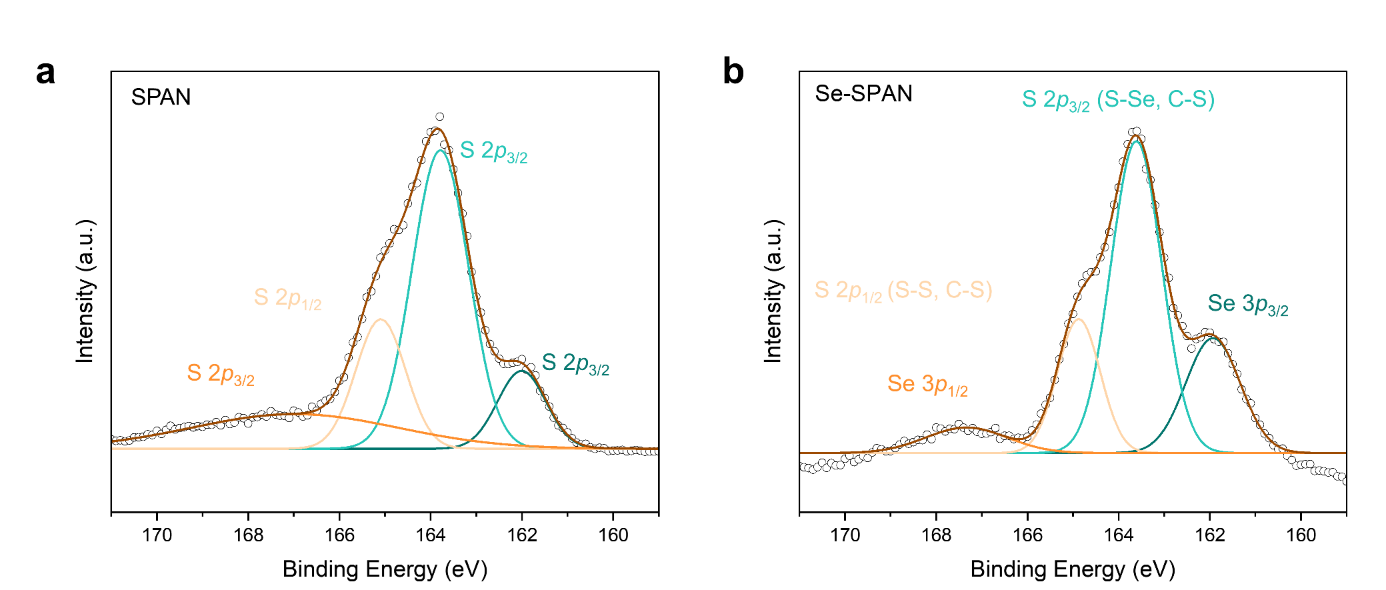


**Figure S1.** **Comparison of SPAN and Se-SPAN.** (a, b) XPS analyses (S 2*p*) of (a) SPAN and (b) Se-SPAN


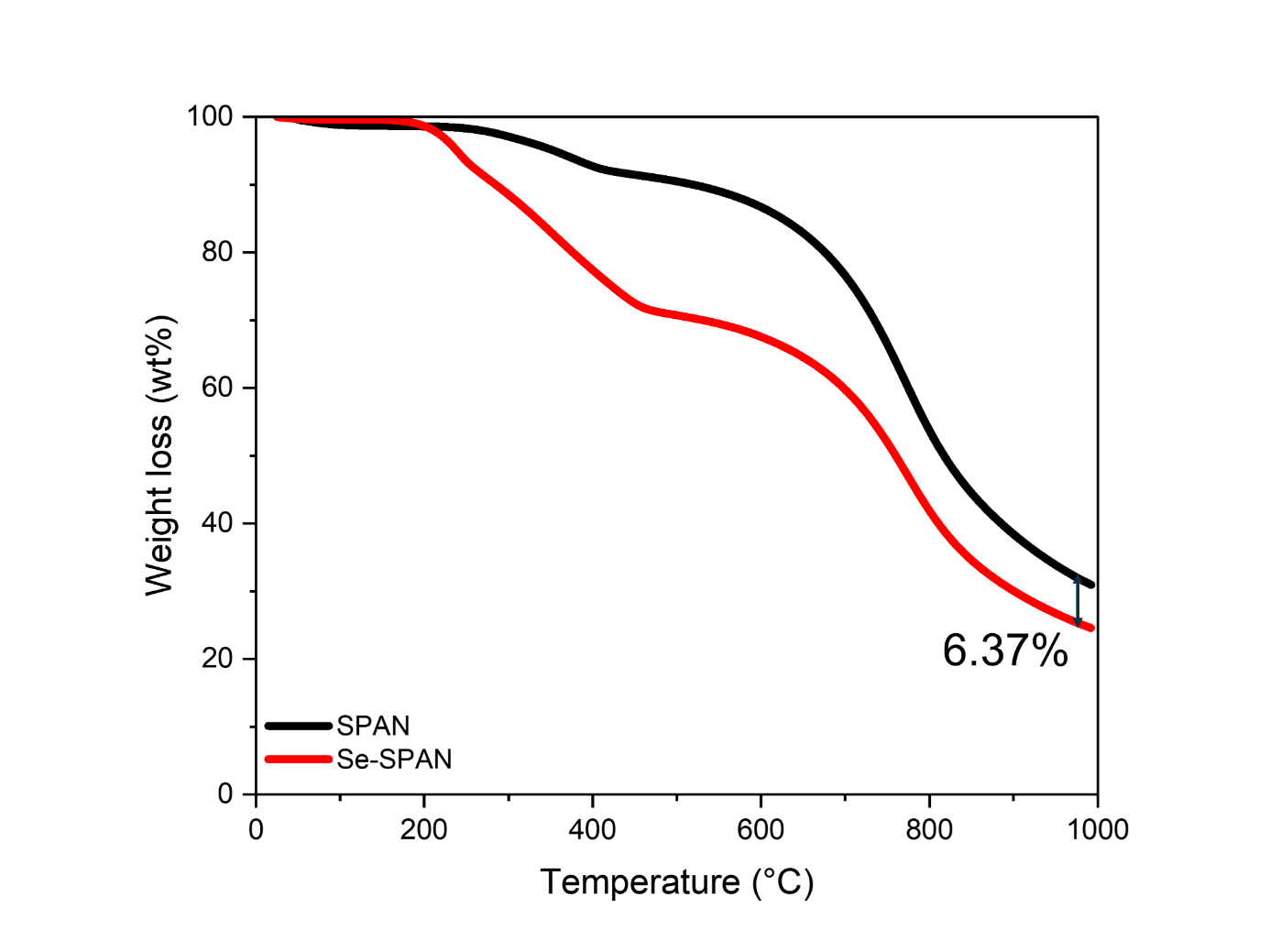


**Figure S2.** **Comparison of SPAN and Se-SPAN.** TGA analysis of SPAN and Se-SPAN.


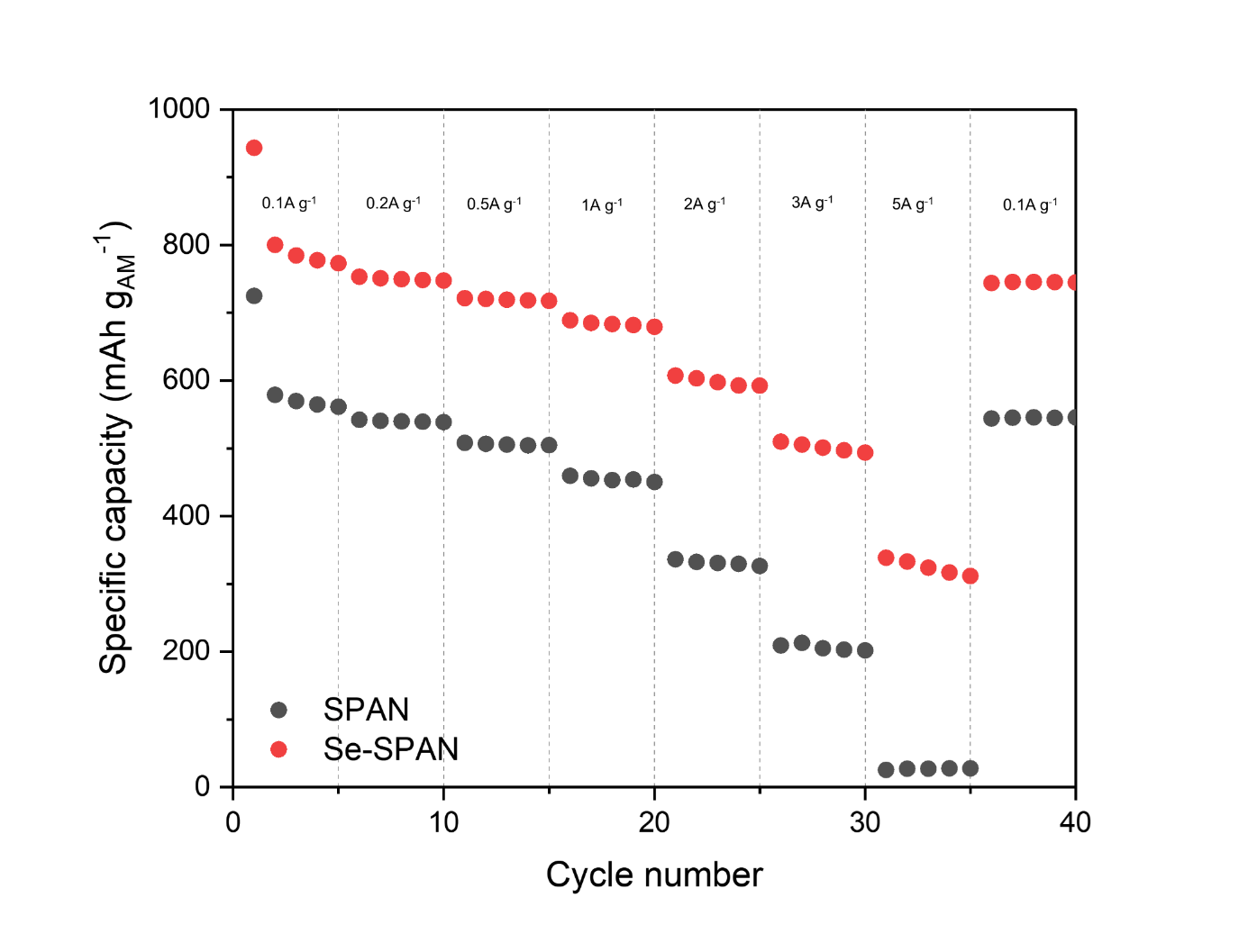


**Figure S3.** **Comparison of SPAN and Se-SPAN.** Rate capability profiles of SPAN and Se-SPAN cathodes fabricated by the slurry process. The areal loading of both cathodes was 0.5 mg cm^-2^.


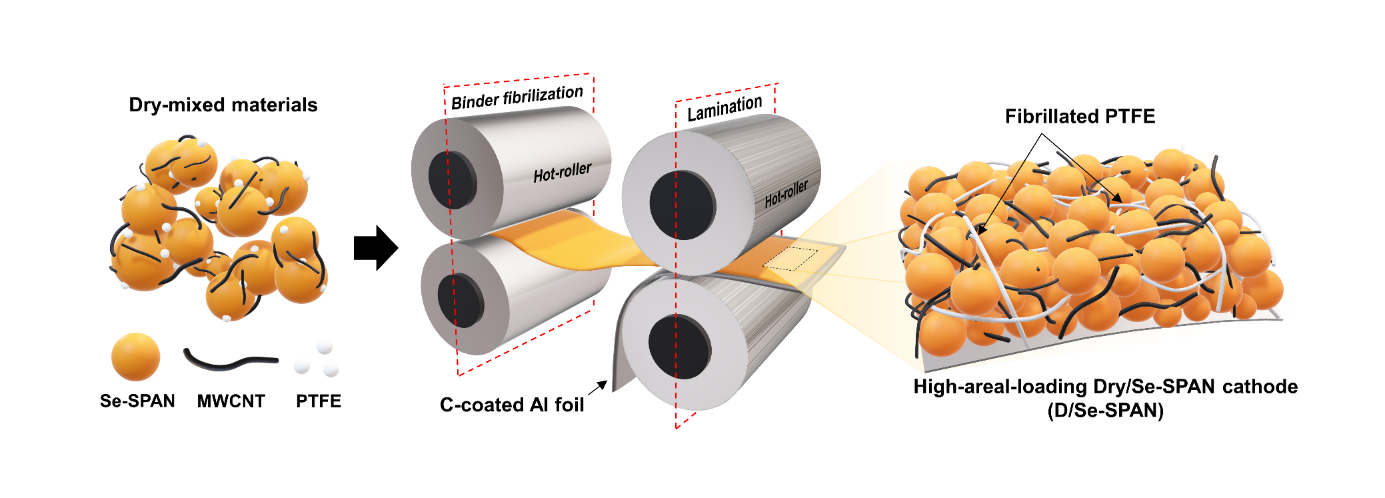


**Figure S4.** Schematic illustration of the fabrication process for the D/Se-SPAN.


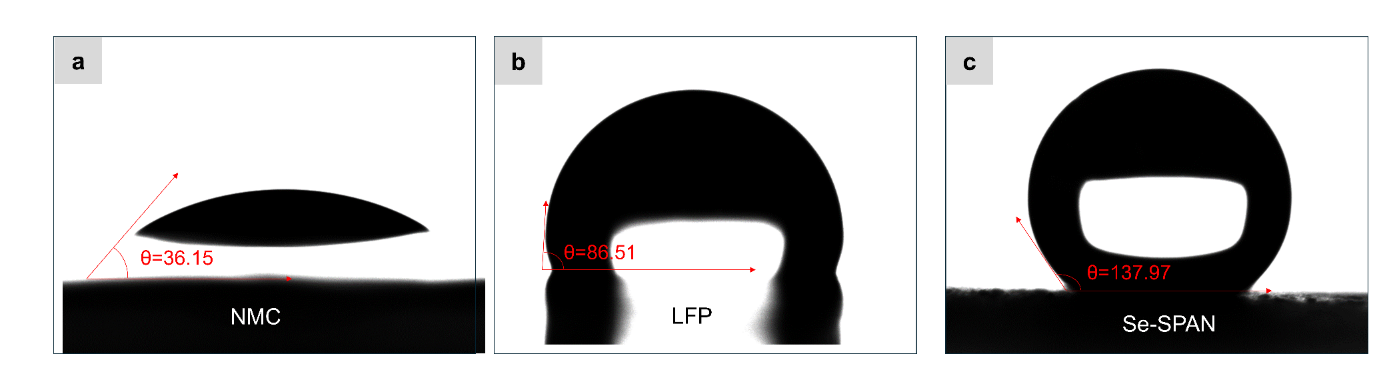


**Figure S5. Materials characterization of NMC, LFP, and Se-SPAN.** Contact angle measurements for (a) NMC, (b) LFP, and (c) Se-SPAN.


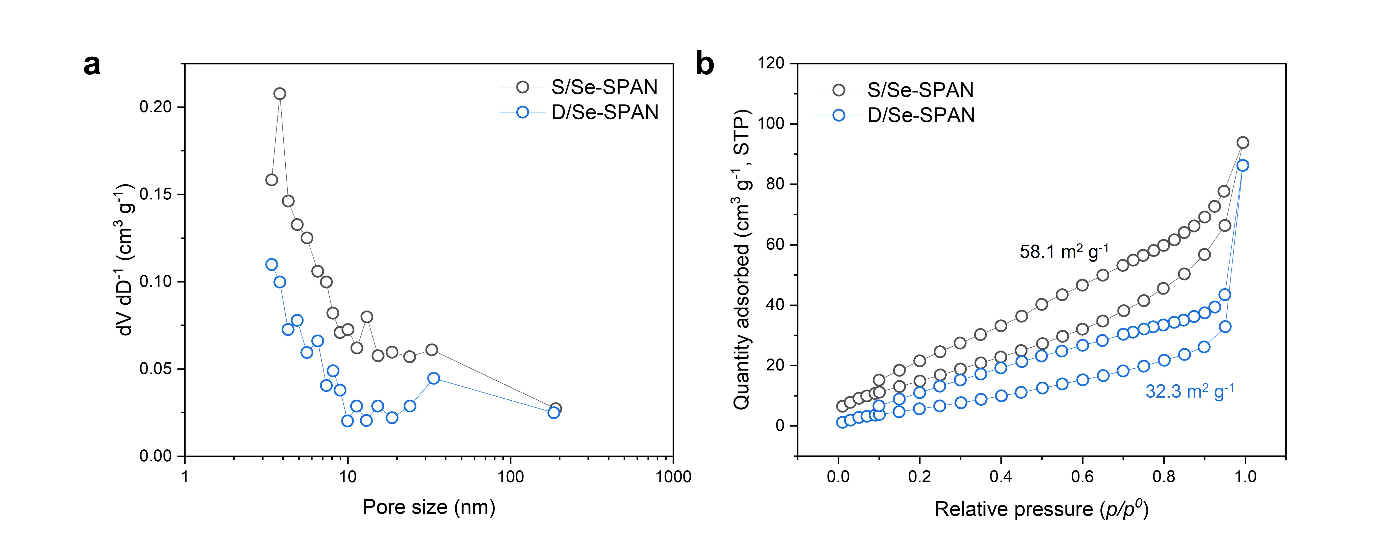


**Figure S6. Pore characterization for S/Se-SPAN and D/Se-SPAN.** (a) BJH plot and (b) Isotherm profiles for both cathodes.


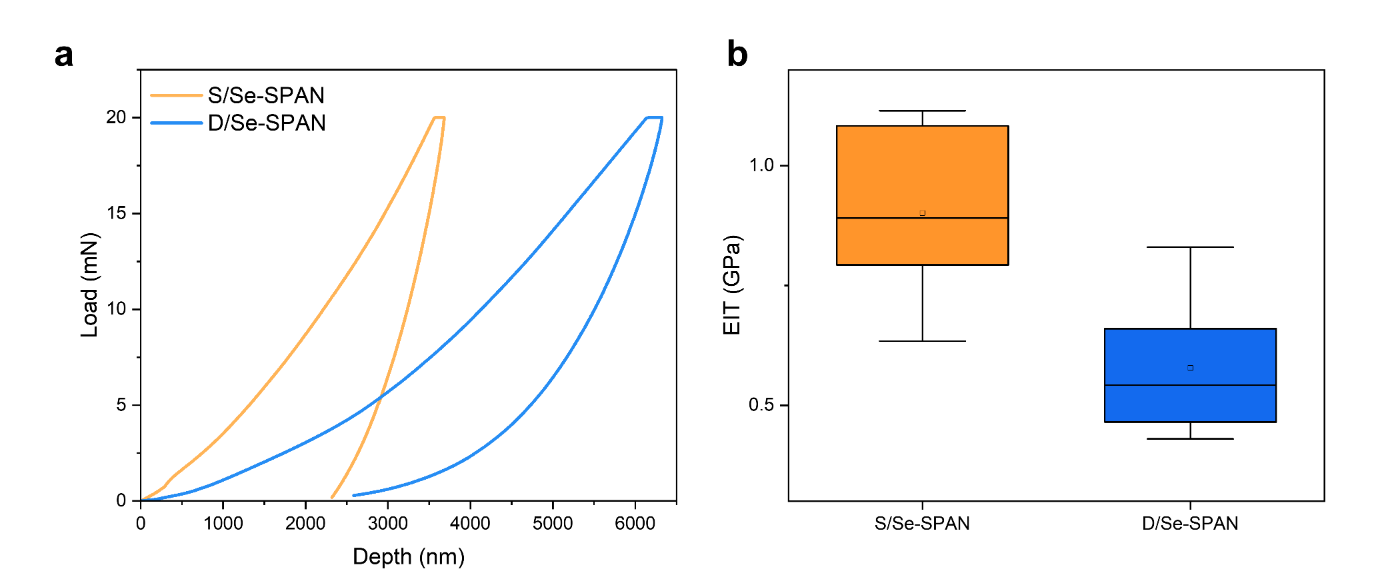


**Figure S7. Materials characterization of S/Se-SPAN and D/Se-SPAN.** (a) load-depth curves from nano-indentation tests comparing S/Se-SPAN and D/Se-SPAN electrodes, (b) reduced elastic modulus (EIT) values for S/Se-SPAN and D/Se-SPAN electrodes.


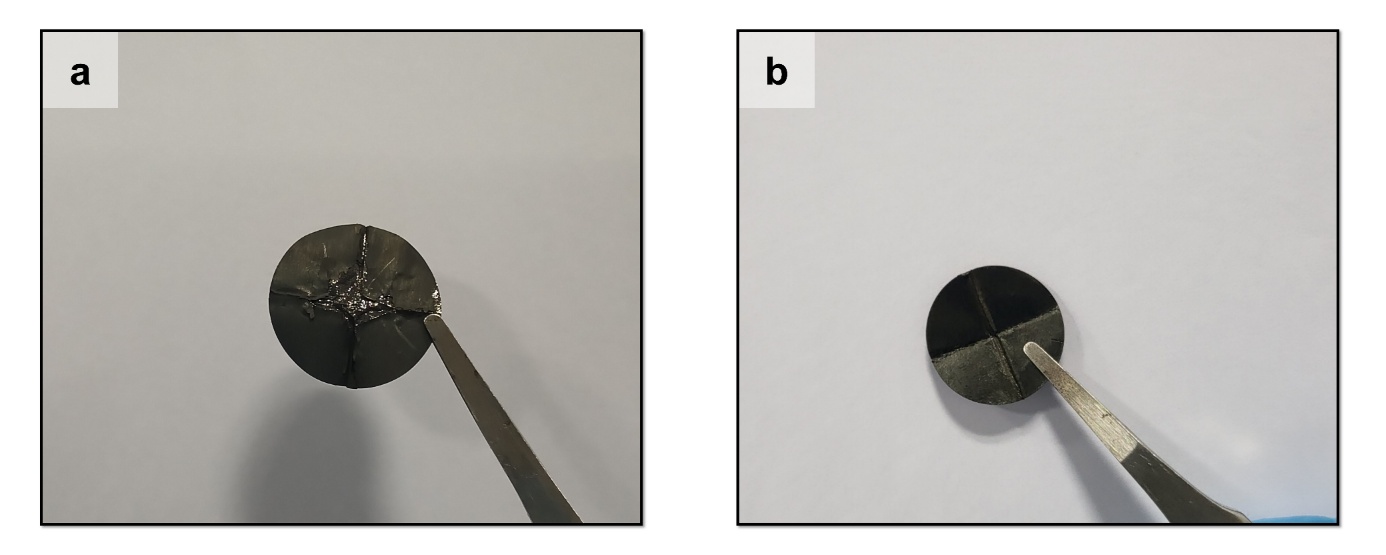


**Figure S8.** Folding test for (a) S/Se-SPAN, (b) D/Se-SPAN.


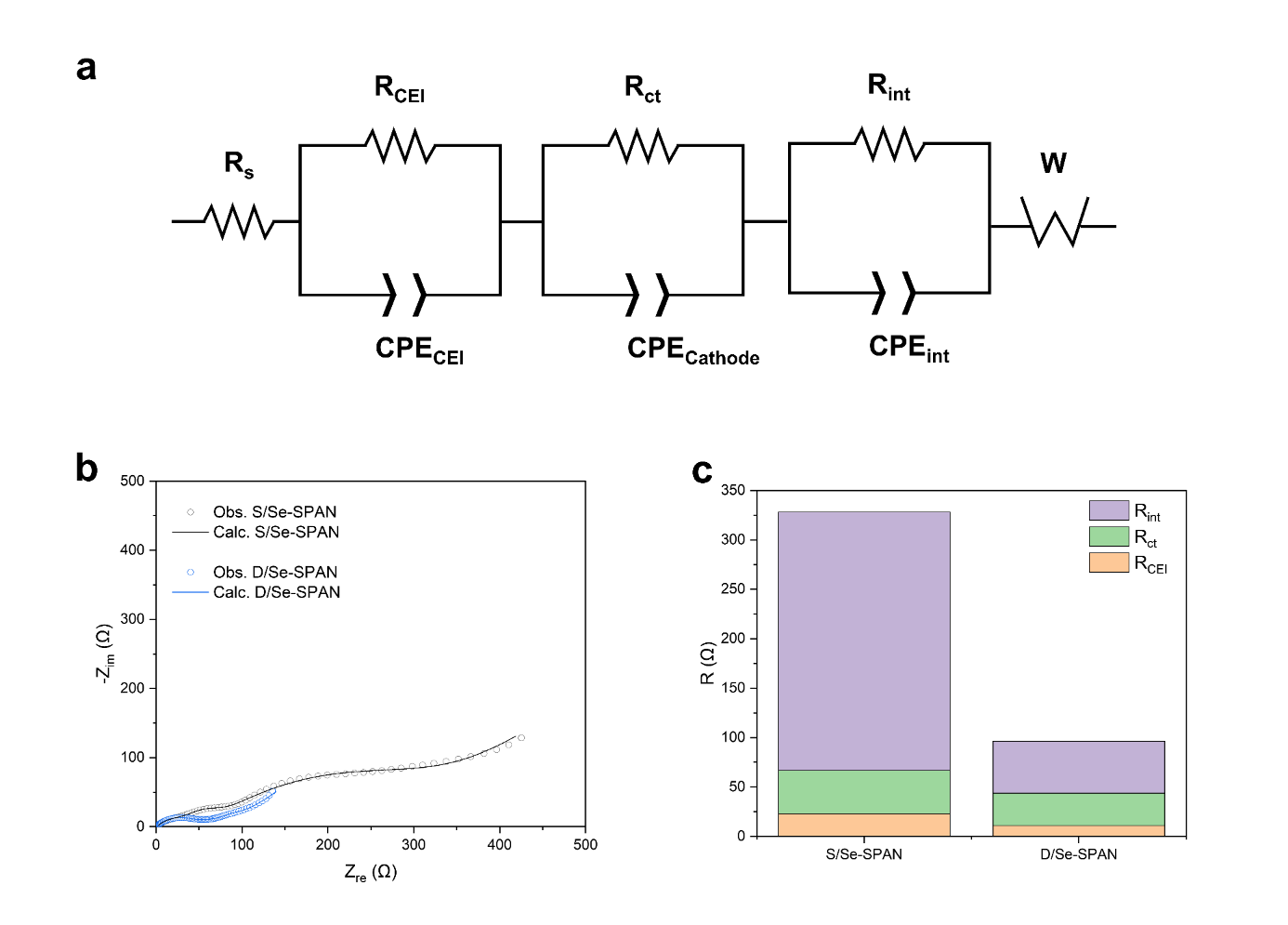


**Figure S9. Evolution of R_CEI_, R_ct_, and R_int_ after formation protocol for both cathodes.** (a) Equivalent circuit model used for fitting the impedance spectra of S/Se-SPAN and D/Se-SPAN cells. (b) Nyquist plots comparing the observed and calculated impedance spectra for S/Se-SPAN and D/Se-SPAN after formation protocol. (c) Dynamics diagram comparing the resistance components (R_CEI,_ R_ct,_ and R_int_) of S/Se-SPAN and D/Se-SPAN.


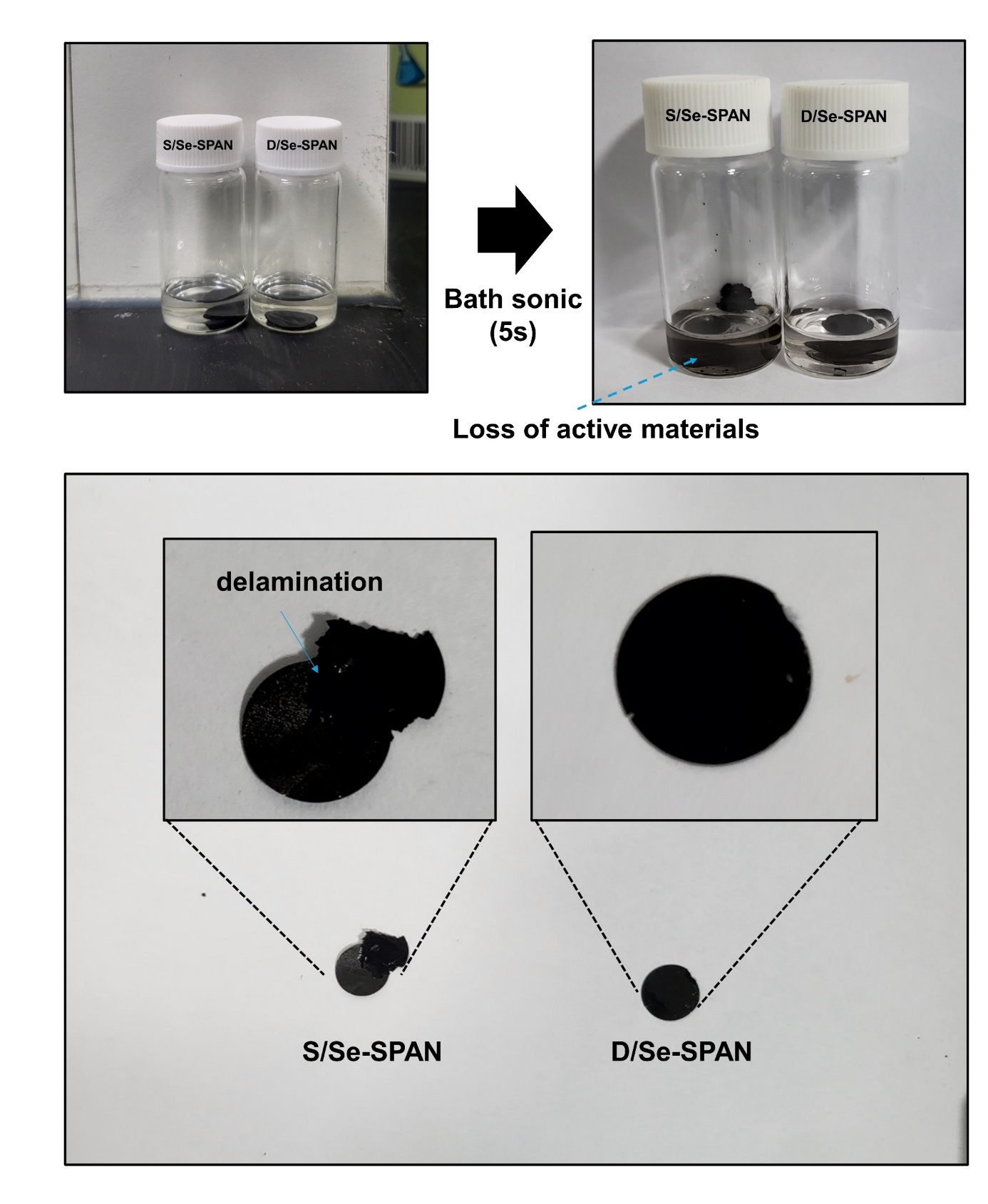


**Figure S10.** Optical images for pre-cycled S/Se-SPAN and D/Se-SPAN after sonication in the electrolyte.


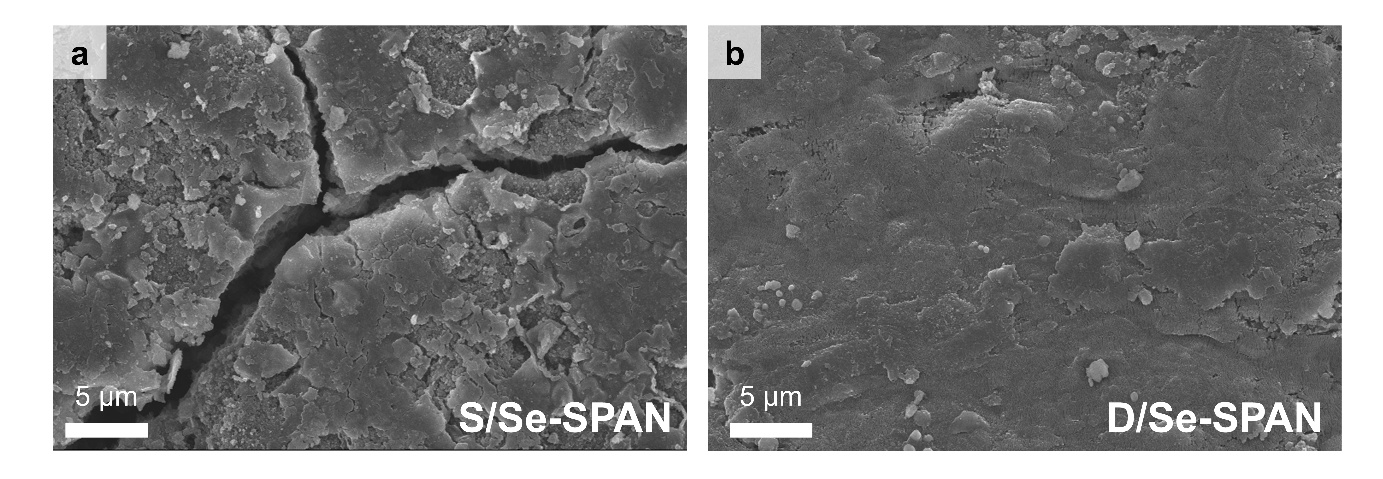


**Figure S11.** SEM images for (a) S/Se-SPAN and (b) D/Se-SPAN after 100 cycles.


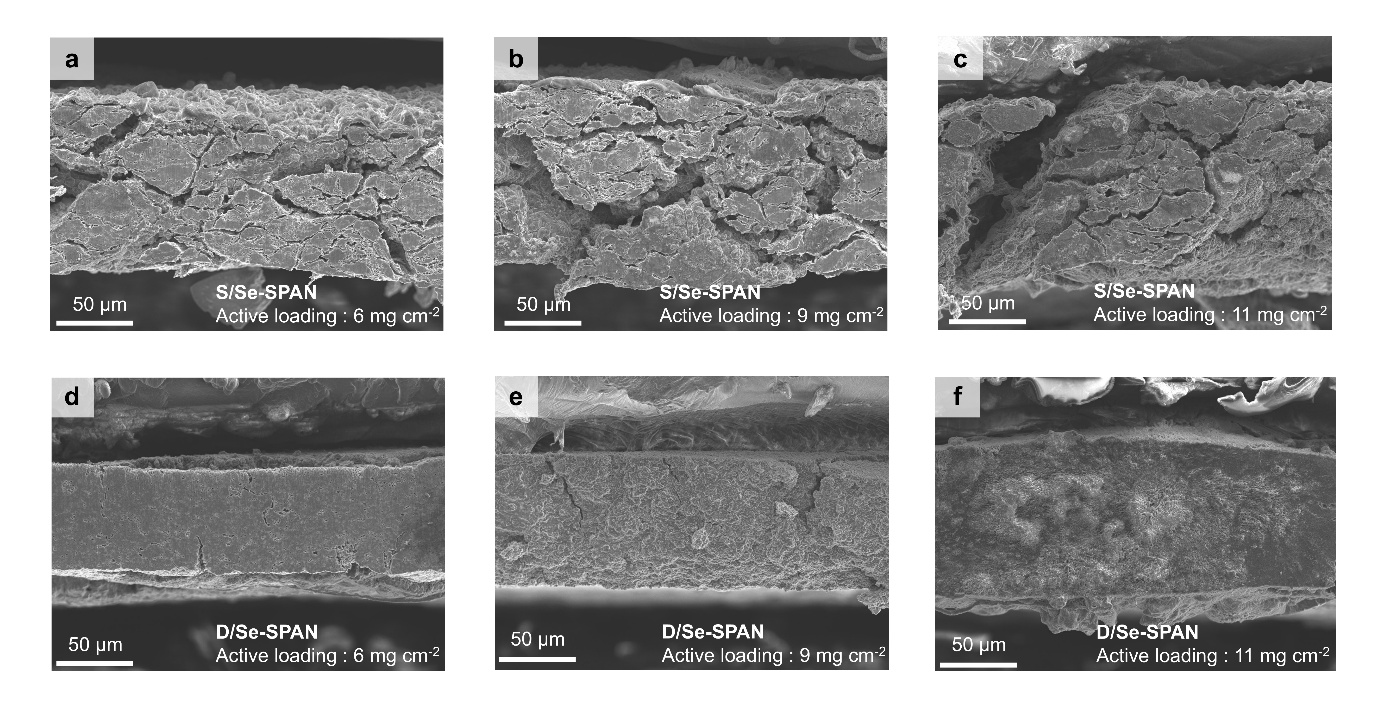


**Figure S12.** Cross-sectional SEM images of (a-c) S/Se-SPAN, and (d-f) D/Se-SPAN electrodes after cycling at different loading levels.


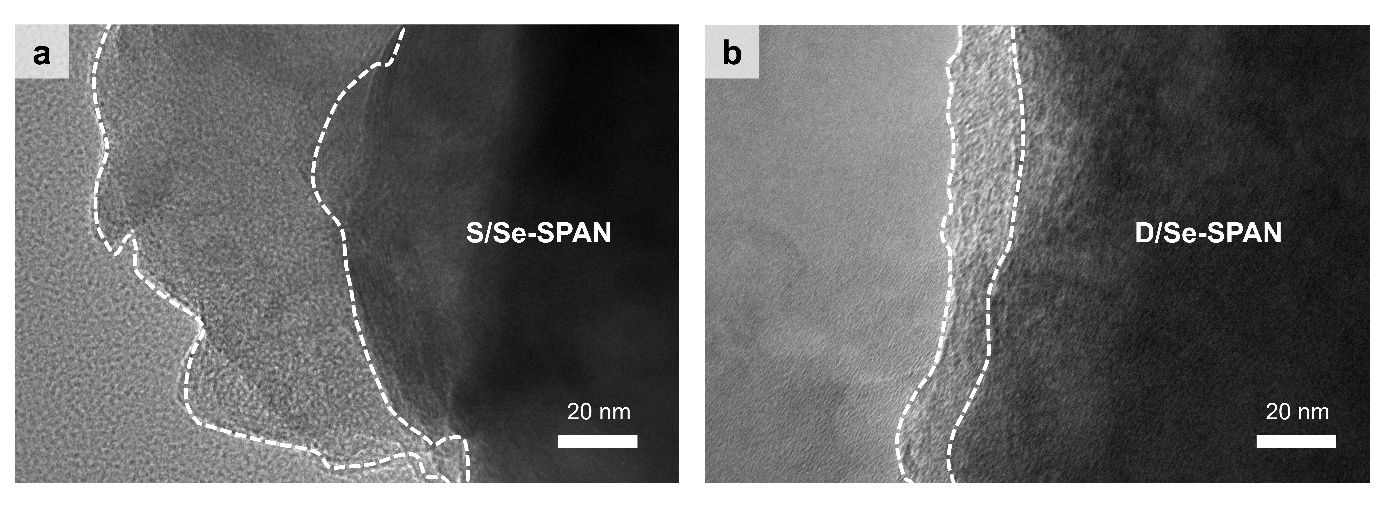


**Figure S13**. TEM images for (a) S/Se-SPAN and (b) D/Se-SPAN after 100 cycles.


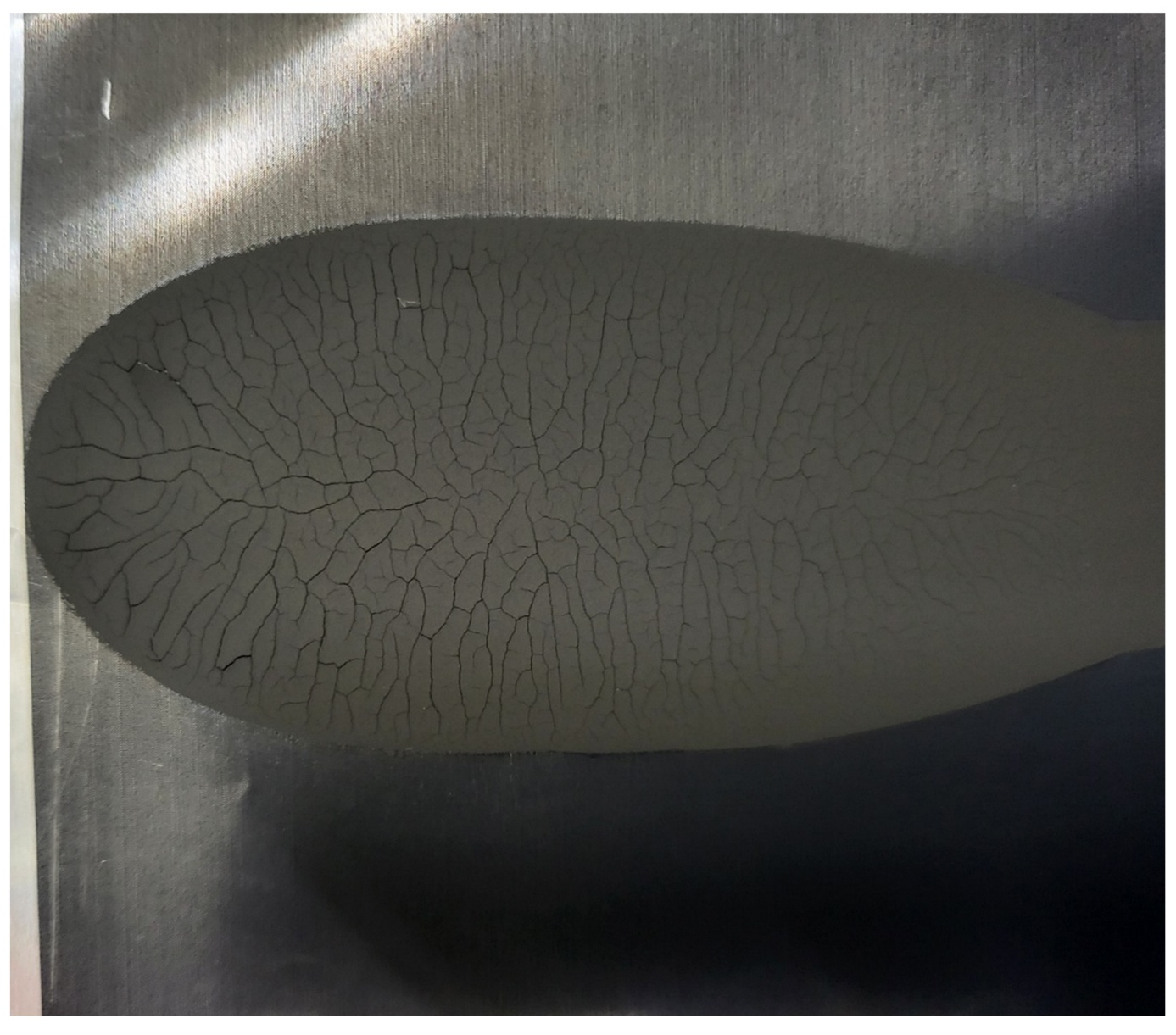


**Figure S14.** Optical image for S/Se-SPAN with high areal loading.


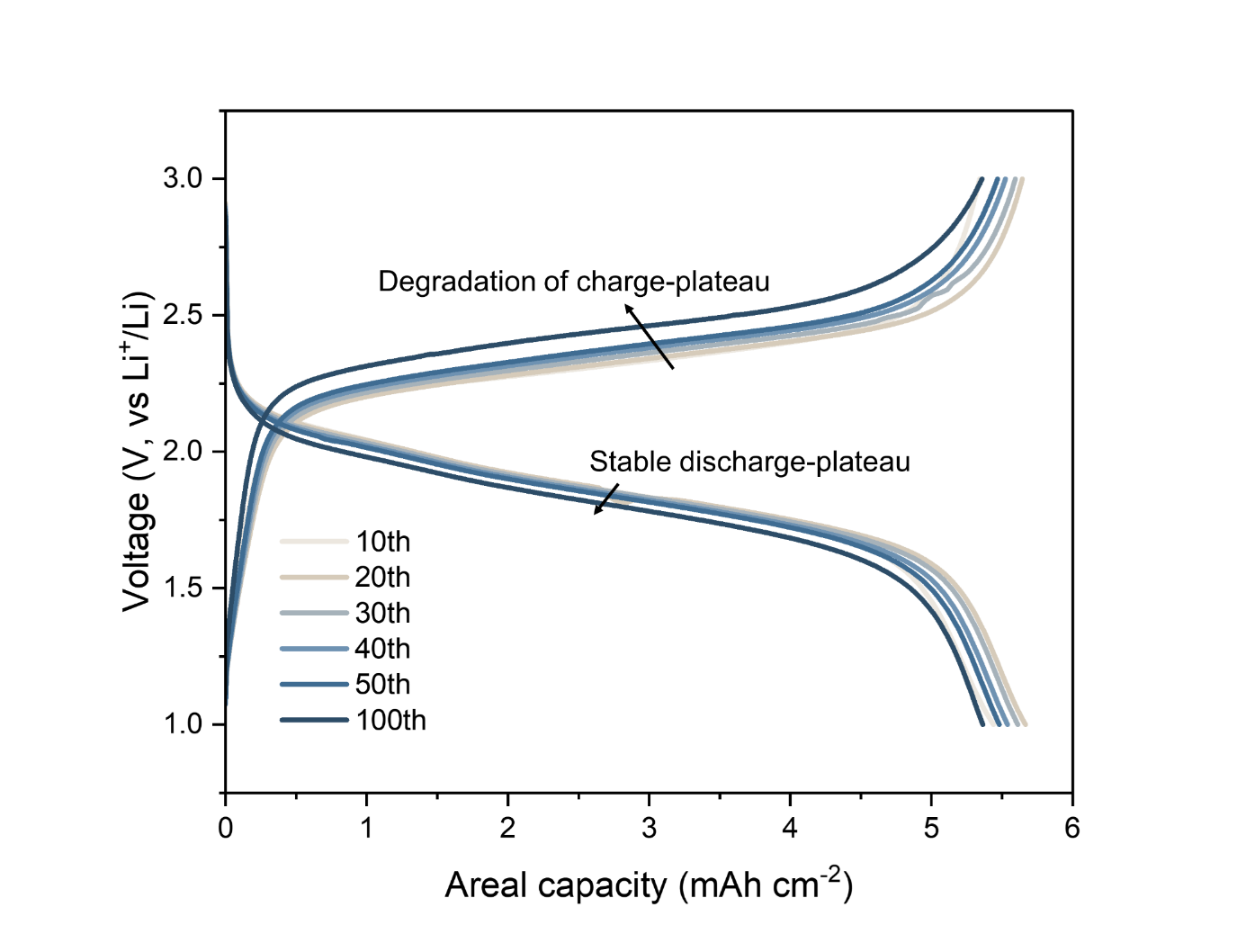


**Figure S15. Charge–discharge profiles of D/Se-SPAN with 9.2 mg cm⁻² during 100 cycles.** The charge–discharge curve illustrate the electrochemical lithium insertion/extraction characteristics of the D/Se-SPAN cell with an areal loading of 9.2 mg cm⁻² with increasing cycle number. Notably, the charge plateau increases with cycling, while the discharge plateau remains stable, indicating that the main factor for voltage degradation originated from issues in the deposition behavior of the lithium anode.


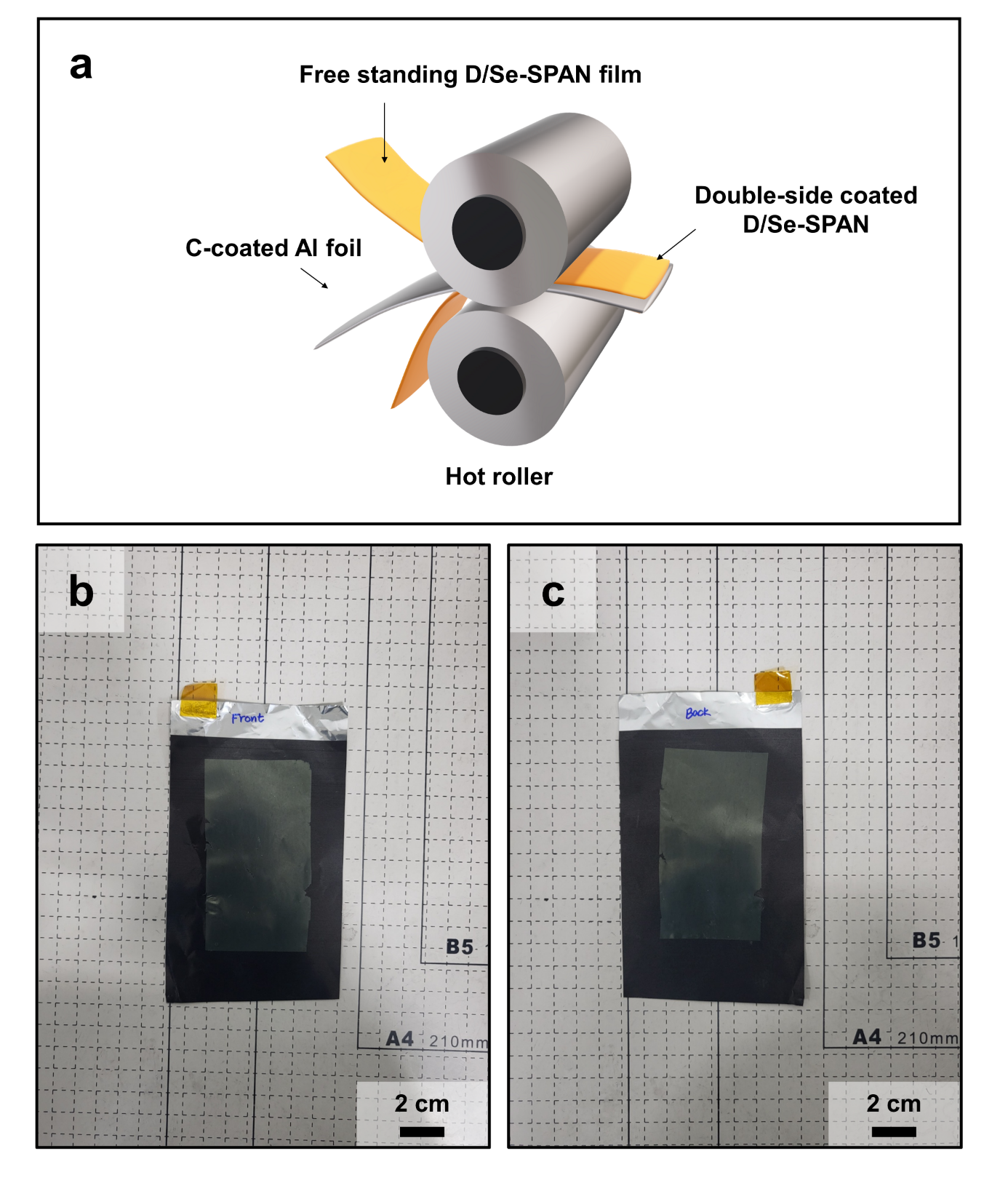


**Figure S16. Dry-process for fabricating double-layer coated D/Se-SPAN.** (a) schematic illustration of the double-layer coating process for D/Se-SPAN. (b) front-side coating. (c) back-side coating.
